# COVID-19 infections and fatalities developments: empirical evidence for OECD countries and newly industrialized economies

**DOI:** 10.1007/s10368-020-00487-x

**Published:** 2020-10-14

**Authors:** Lucas Bretschger, Elise Grieg, Paul J. J. Welfens, Tian Xiong

**Affiliations:** 1 ETH Zurich, Zurich, Switzerland; 2 EIIW/University of Wuppertal, Wuppertal, Germany

**Keywords:** Coronavirus pandemic, Fatality rates, Air pollution, OECD countries, Newly industrialized countries, Health systems, Environmental policy, F63, H12, I10, I18, Q53

## Abstract

This paper presents empirical results on coronavirus infection and fatality rates from cross-country regressions for OECD economies and a sample of middle- and high-income countries. We include environmental, economic, medical, and policy variables in our analysis to explain the number of corona cases and deaths per million. We find a significant positive impact of local air pollution on infection rates in the whole sample and on fatality rates for OECD countries. Obesity rates have a positive effect on cases and deaths across the different estimation equations. The strategy of aiming to achieve herd immunity has a significant positive effect on infections as well as on death rates. The first affected countries have significantly higher mortality rates, revealing the lack of experience and medical capacity to deal with the pandemic in an initial phase. Postponing – and fighting - the pandemic could save lives in many countries and generate considerable economic benefits. Other medical and policy variables discussed in the public sphere do not show a significant impact in the regression analysis. Our results suggest that improving air quality and fighting obesity helps reduce the negative effects of a coronavirus pandemic significantly. Policy options for fighting a second epidemic wave should take into account the results from this study in order to optimize global epidemic policy.

## Introduction

The coronavirus pandemic is a historical challenge for the world economy – in medical as well as economic and political terms. To achieve an understanding of the numbers of infections (cases) and fatalities from the novel coronavirus, it is crucial to have an adequate analytical framework and to come up with significant empirical results which will, of course, be relevant for economists, the business community, medical researchers and policymakers worldwide. One straightforward analytical approach is to start with a simple consideration: Negative pollution externalities are a key topic of environmental economic research - diseases, particularly communicable diseases, are another important form of negative impact on both human well-being and the economy at large. They can cause great damage, especially when they occur on a large scale, such as the recent coronavirus pandemic and the associated disease known as COVID-19.^1^ After its emergence in China in December 2019, the disease quickly spread around the whole world. Within a few months, governments around the globe have taken measures to combat the epidemic in their own countries – including temporary lockdowns of the population and shutdowns of certain production activities. The rapid spread of the virus in Western Europe and the US has presented an enormous test for acute care stations in hospitals where, in April and early May 2020, capacities were fully exhausted in some regions of Italy, France, Spain and the UK.^2^ The novel coronavirus and the disease which it has caused was initially considered to be a “pneumonia of unknown etiology” and early research identified that the underlying virus was related to the coronavirus grouping, possibly related to SARS and MERS (Sun et al. 2020).^3^

Successfully fighting the pandemic is of high economic relevance as the global economic costs have been estimated to be $200 billion per week and about $80 billion per week for the US alone, namely in the form of foregone production and extra expenditures in terms of health care system (Summers 2020). To the extent that the subsequent empirical results presented allow, in principle, to postpone the global diffusion of COVID-19 infections and thus the number of COVID-19 deaths, respectively, by at least two weeks via adequate policy measures worldwide, a combined international epidemic policy therefore would bring a global economic welfare gain of about $400 billion – almost 1% of global Gross Domestic Product (GDP). Thus, expansionary fiscal policy could, in turn, be reduced by about 1% of GDP (assuming a multiplier in the order of about unity) which would bring a lower increase of the debt-GDP ratios of OECD countries and Newly Industrialized Countries as well as developing countries. The ADB has estimated that global economic losses from the pandemic could be in the range of $4.1 trillion to $5.4 trillion whereby economic policy intervention has been assumed to have mitigated the output loss by 30–40%. As a region, Asia (the apparent origin of the pandemic) is expected to account for about 30% of the global output loss (ADB 2020). This is the economic perspective of the subsequent empirical COVID-19 analysis – with several key economic policy implications picked up in the final section.

In the spring of 2020, it became rather clear that COVID-19 is often associated with a broader range of problems for the infected persons, namely an Acute Respiratory Distress Syndrome (ARDS). From this perspective, it is of particular interest to understand how existing respiratory problems in certain patients and the state of the environment in the form of air quality problems could possibly contribute to morbidity and mortality, respectively; this would establish a direct link between the external effects of pollution and pandemics. Other patient predispositions, such as obesity or diabetes, could also play a role. Autopsies carried out by medical researchers in Basel (Switzerland) and Hamburg (Germany), for example, have revealed that in many COVID-19 deaths, evidence was found of a critical role played by the predisposition of patients and other health problems which in the end can make the COVID-19 infection a deadly infection (ARD 2020).

Since the coronavirus pandemic stands for a novel – and rather aggressive – virus, it is clear that the availability of high quality hospital facilities, including acute care stations, could play a key role in dealing with the spreading of the virus**.** As regards the quality of national hospital systems, there is no clear international indicator system available with the exception of the resistance problems of patients treated in countries with a lack of differentiation in the usage of antibiotics in hospitals: MRSA-related problems (MRSA = Methicillin-Resistant *Staphylococcus aureus* is a group of bacteria that are genetically distinct from other *Staphylococcus aureus*. MRSA often is considered to be responsible for several difficult-to-treat infections amongst patients in hospitals), for example, are known to be a rather serious challenge in many hospitals; MRSA statistics could indeed be considered to be an indicator of the overall quality of national hospital systems. There is a rather short history of international comparative research on MRSA problems in hospitals (e.g., Aliberti et al. 2016). With many patients having to be admitted to hospital in the early stage of the pandemic, such structural weakness points could add to COVID-19 fatality rates.

The subsequent empirical analysis takes into account many variables in an effort to explain fatality rates; herein, some of the regression results with the most interesting findings will be presented for OECD countries as a sub-group; regression results for the overall group of countries and the Newly Industrialized Countries (NICs), respectively, are also are quite interesting and show differences across the two groups of countries. The group of OECD countries is of particular interest since many OECD countries were reaching a peak in infections and fatalities in a rather parallel fashion; but there is also the differentiation between those countries which aimed rather at achieving an early level of herd immunity – notably, Sweden, the UK and the Netherlands – and other countries which placed more emphasis on quarantine measures and social distancing as well as other selective interventions with the aim of minimizing the diffusion of the coronavirus.

Besides the historical medical challenge, COVID-19 infections have created serious economic problems in more than 100 countries, in particular in OECD countries where the output decline in the first and second quarters of 2020 has reached double digits. The IMF (2020b) has forecasted in its World Economic Outlook that world output will decline by 4.9% in 2020 – followed by a growth rate of 5.4% in 2021; and that both OECD countries and newly industrialized countries will face serious recession pressure. The World Bank’s analysis (World Bank 2020) has suggested that in the context of the corona shocks about 90 countries could face an output decline in 2020, an historical situation not seen since 1870. One may hope that some OECD countries could manage to achieve a fast and strong economic recovery. Even if one would follow the scenario analysis of the Bank of England (2020) that the UK will have a 14% output decline in 2020, followed by a 15% increase of output in 2021, the Bank’s warning that the United Kingdom might witness the worst recession in 300 years naturally is a cause for concern. The impressive growth which was witnessed in China over many years came to a halt in the first quarter of 2020 when Chinese authorities were coping with the COVID-19 challenge, which seems to have emerged early on in the province of Hubei at the end of 2019. In the US, the number of unemployed has increased by more than 40 million within only twelve weeks. For certain OECD countries, the enormous expected output declines, the steep rise of deficit-GDP ratios, and the strong increase of unemployment figures (IMF 2020a, b; European Commission 2020; Pfeiffer et al. 2020) indicate an enormously negative side-effect of the coronavirus pandemic.^4^ While the earlier SARS and MERS epidemics where primarily regional, from an international perspective, the coronavirus pandemic is truly global and a very serious medical, social, political and economic challenge for most countries. From an economic perspective, the coronavirus pandemic is in the first instance a global symmetric shock, however, different reactions of policymakers in various countries could create differing epidemic developments across countries. The IMF World Economic Outlook of April 2020 suggested that the world economy will face an almost global recession (IMF 2020a).^5^ The IMF’s WEO update of June 2020 suggested further weakening of international output forecasts (IMF 2020b).

The present paper provides empirical evidence on the effect of pollution on COVID-19 fatality rates in middle- and high-income countries. It relates to various strands of recent literature. An early publication on the economic and health care aspects of the coronavirus pandemic is Welfens (2020a) who points to the role of health system quality and identifies theoretical aspects related to growth modelling and the structural breakdown of the economy.^6^ Holtemöller (2020) develops a medium-term economic model in which an epidemic model is combined with an economic business cycle model.^7^ The relationship between health and the environment has been the subject of a specific literature. In an early contribution to the theory, Gutierrez (2008) uses an overlapping generations framework where pollution imposes health problems on households when they are elderly; pollution raises health costs inducing precautionary savings and capital accumulation so that the economy is more likely to be dynamically inefficient. In a similar setup, Wang et al. (2015) study precautionary savings, health insurance, and environmental policy as a response to health risks, which depend on environmental pollution; it is found that optimal environmental policies and the optimal health insurance environment are deeply intertwined. Bretschger and Vinogradova (2017) develop a stochastic framework for an endogenously growing economy, which is subject to pollution-induced health shocks and where the health status is a component of the welfare function. The paper derives closed-form analytical solutions for the optimal abatement policy and the growth rate of consumption; it shows that devoting a constant fraction of output to emissions’ abatement allows for achieving the first-best allocation in the economy. Bretschger and Vinogradova (2019) generalize the concept of induced shocks to a broader class of models for endogenously growing economies and derive optimal policies to reduce the damage to households efficiently.

Turning to empirical studies, early data from case fatalities in China suggested that the elderly population experienced a higher mortality rate than the overall population (Wang et al. 2020). With respect to coronavirus-related deaths in the US, there is an early empirical analysis of case fatalities by medical researchers for US regions (Wu et al. 2020). The authors consider a battery of medical and other variables to explain regional case fatalities in the United States.^8^ Sherpa (2020) looks into the specific role of austerity policies on COVID-19 fatality rates and indeed finds significant evidence in the case of OECD countries for that variable. Sherpa’s quantile regression analysis indicates that austerity measures in OECD countries (here, cuts to health expenditures) significantly increase the COVID-19 mortality rates in those countries. Early US medical research has pointed to the role of air quality problems for regional fatality ratios in the US (Wu et al. 2020). The COVID-19 fatality ratio is a much more serious indicator of the effects of the epidemic; the lower the average age of death, the more (hypothetical) lifetime losses are incurred – here, the US has witnessed a lower average age of death than the EU countries in the first half of 2020 (Economist 2020).

As regards COVID-19 morbidity and mortality, the medical and economic challenges in the first half of 2020 have not only been faced by the OECD countries but by NICs as well. To the extent that NICs are also included in the research, as is the case in the subsequent analysis, it is clear that the average age of death is likely to be lower in NICs than in OECD countries where one can see a higher median age and a higher share of people above 65 years: a higher age of patients is expected to go along with a higher mortality rate for many infections and COVID-19 is a key disease here. If one is looking for statistical correlations for fatality ratios and medical variables, one should not overlook the potential case of paradoxical mortality linkages: Countries with a high share of cardiovascular patients prior to the corona shock year 2020, for example, are likely to have more intensive care units (ICUs) in hospitals than other countries – this would then be of help to such countries in facing the coronavirus epidemic as the overall number of ICUs is higher than in countries with a low share of cardiovascular patients. Subsequently, such a correlation is indeed identified and with the prevalence of diabetes, a similar link could be relevant. There are other variables which one could consider in the context of the broad perception that COVID-19 will typically seriously impact the respiratory system of infected patients: the share of smokers for instance is a variable that is sometimes discussed but, as will be shown, there is no empirical evidence of such a link.

To the extent that the limited number of OECD member countries (i.e., 37 if the recent accession country Colombia is included) – with observations in the first half of 2020 - is a problem for empirical cross-country analysis, one can consider a broader sample of countries which should include mainly NICs for which broad data are available. This is the strategy adopted in a separate subsection below which indeed identifies several medical, demographic as well as economic drivers of infections for the broader sample of countries. Moreover, as regards the narrower OECD country sample, it is possible to identify significant variables for the COVID-19 fatality ratio; these include the impact of herd immunity strategies which also turns out to be significant in the broader sample of countries. One may emphasize that by mid-2020, both OECD countries and most NICs had reached their respective national peak of COVID-19 mortality rates, while in many developing countries mortality rates still showed a clear upward trend in the WHO data (see various daily summaries of the WHO situation reports, e.g. WHO 2020a, d, e). As regards the COVID-19 incidence in terms of both morbidity and mortality, it seems that certain OECD countries were ahead of the NICs, with the exception of Brazil which, however, is a special case since President Bolsonaro had adopted a herd immunity strategy at the outset of the coronavirus pandemic. A herd immunity strategy lets one expect higher case numbers than in countries without such a strategy; however, one should also not rule out higher fatality ratios (i.e., the number of COVID-19 deaths per million inhabitants) since the herd immunity strategy could lead to an overwhelming of the capacities of intensive care units in hospitals in some or many regions – an increase of the fatality ratio will be the consequence.

As regards the structure of the respective underlying virus, SARS, MERS and COVID-19 are closely related. With respect to the link between pandemics and the state of the environment, Cui et al. (2003) report a positive association between air pollution and SARS case fatality rates in the Chinese population studying 5 regions with 100 or more SARS cases. Evans and Smith (2005) examine whether serious health conditions are related to current and long-term exposure to particulate matter and ozone. The findings suggest significant current and long-term effects of air pollution exposure on new cases of heart attack, angina, chronic lung conditions, and shortness of breath. He et al. (2016) study the exogenous variations in air quality during the 2008 Beijing Olympic Games and find that a 10% decrease in PM10 concentrations reduces the mortality rate by 8%. Deryugina et al. (2019) estimate the causal effects of acute fine particulate matter exposure on mortality, health care use, and medical costs among the US elderly using Medicare data. They use changes in local wind direction as an instrument and machine learning to estimate the life-years lost due to pollution exposure. The paper finds that mortality effects are concentrated in about 25% of the population of elderly residents. In a quantitative cohort study conducted between 2000 and 2018 in six US metropolitan regions, Wang et al. (2019) find that long-term exposure to ambient air pollutants is significantly associated with increasing health problems in particular emphysema and worsening lung function. Summarizing previous empirical findings, Conticini et al. (2020) conclude that individuals living in areas with high levels of air pollution are more prone to developing chronic respiratory conditions, which partly explains a higher prevalence and lethality of novel, highly contagious, viral pandemics such as COVID-19 in those regions.

Our paper builds on these contributions and tests the main empirical hypotheses with novel data for COVID-19 fatality rates in different sets of countries. The empirical analysis on the country level has to consider the heterogeneities between the countries which we accommodate by inclusion of appropriate control and dummy variables. We chose the OECD countries as our first sample because these economies are quite similar in basic aspects of development, institutions, and COVID history. Our second sample includes Newly Industrialized and other Middle Income Countries, which increases the number of observations. We highlight that a study at country level offers a number of advantages compared to a study at regional level. First, one key variable of concern, air pollution, has a larger variation for countries which benefits the accuracy of the results. Second, pollution is mainly driven by policy choices, e.g. concerning energy and transportation systems, which are mainly taken at the national level. Political decisions are exogenous in our setup so that the need for instrumenting the pollution variable is not imminent. Third, we are able to study the impact of pollution jointly with the effects of health status, health and other policy, as well as economic conditions, which are all determined on the country level providing a broader perspective than analyses for single countries. Key drivers of infections are per capita income and air quality problems, while main drivers of fatality ratios in OECD countries are air pollution, obesity and the herd immunity variable; slowing down new infections and the spreading of the coronavirus can save lives – if our findings for 104 countries would apply to the overall world economy, a one week postponing of infections would save 183,624 lives globally.

In the subsequent analysis, we first take a closer look at measurement aspects of infection cases and fatality rates in OECD countries as well as in selected NICs (Section 2). Section 3 develops the basic hypothesis for the subsequent empirical models and describes the data series. In Section 4, we present the regression results for OECD countries and, separately, for the larger country sample. Section 5 concludes with policy conclusions and perspectives for further research.

## Corona case fatalities: Descriptive statistics and data problems in an international perspective

The basic idea, based on the previous discussion and the literature, respectively, is to analyze the link between case fatality rates related to the novel coronavirus and a selection of exogenous variables which should include medical, demographic and environmental factors plus other data. As a first step, one has to consider the measurement of fatalities from COVID-19 where several varying sources and methodologies exist.

There are three different approaches to measuring fatalities from COVID-19 cases, namely (i) the Johns Hopkins University (JHU) approach covering different data sources (JHU 2020), (ii) the WHO measurement approach based on the official governmental reports of the member countries, and (iii) the excess mortality estimates that indirectly attempt to measure COVID-19 deaths. For (ii) we have to note the differences in the measurement of COVID-19 deaths between different regions and institutions, even within individual countries. For (iii), excess mortality figures are available from EuroMOMO, which is a network covering 24 countries/regions in Europe.^9^ One important policy perspective here could be to assess the need for international and intra-country (regional) political solidarity based on excess case fatalities if there are different international or regional classifications/coverage of COVID-19 fatalities.^10^ The concept of excess fatalities, i.e. the difference between the actual numbers of deaths in a certain period compared to the number one could normally expect for the same period could be a useful measurement tool for covering COVID-19 fatalities in an international environment in which countries’ COVID-19 fatalities statistics are not harmonized. There is, however, the problem of data availability and indeed a need that the OECD and the UN would provide harmonized excess mortality statistics.^11^

Additionally, national statistical coverage might be different at the beginning of the epidemic and in the later peak stage where for practical reasons the coverage could change; e.g., with acute care capacities in hospitals overwhelmed and a lack of sufficient testing kits available, the testing for COVID-19 patients who die at home or in care homes will be rather incomplete at that particular stage of the epidemic. If countries are all close to or immediately beyond peak fatality – with a logistical curve relevant for infections and case fatalities, respectively – no major problem with a comparative analysis of case fatalities should occur since countries’ fatalities and case fatality rates are in the upper, flat, part of the logistical curve. In the EuroMOMO bulletin for week 18 (late April 2020), the authors note for the European countries covered: “The excess mortality estimated by the EuroMOMO over the past weeks appears to have peaked in all countries by now.” (2020a, p.1). From this perspective, a regression analysis of cumulated case fatalities in western and eastern European countries at the end of May should be adequate; one may also assume that the US peak in case fatality rates had been achieved in May 2020. To the best knowledge of the authors, no OECD country is still expecting a peak in case fatality rates in summer 2020.

As regards the number of infected persons, the WHO and the Johns Hopkins University coronavirus research group (Dong et al. 2020) report slightly different numbers of COVID-19 case fatalities.^12^ Differences are explained by the fact that the WHO relies on national governments’ reported fatality numbers while the Johns Hopkins University also takes into consideration press reports on case fatalities (JHU 2020). All reported data naturally contain a lag of about a week since testing and test result reporting as well as death reporting brings delays. Our subsequent analysis will, however, not look at the death rate of a single day – as reported by authorities, the WHO and the JHU, respectively; rather we are interested in explaining the cumulated case fatalities associated with COVID-19. To the extent that epidemics typically follow a logistical curve – with the number of patients recovering (R’; assumed to have immunity against the virus) being a barrier to the further spread of infections - there is a theoretical problem in comparing death rates across countries to the extent that the start of the respective national epidemics show large lags across countries. As regards lags in OECD countries, one may assume that the enormously dense flight and travel networks, respectively, will bring smaller time lags across countries. It should also be mentioned that as long as the absolute number of infections is small, the contact tracing of infected persons is obviously relatively easy so that an early detection of the outbreak and massive tracing and quarantine measures could strongly bend down the infection curves – see, e.g., the Republic of Korea and Taiwan. In the OECD countries, only Iceland appears to be a country where early testing and government intervention seems to have brought a particularly favorable situation in terms of infection intensity (infections – as officially measured – relative to population).

**Fig. 1:**
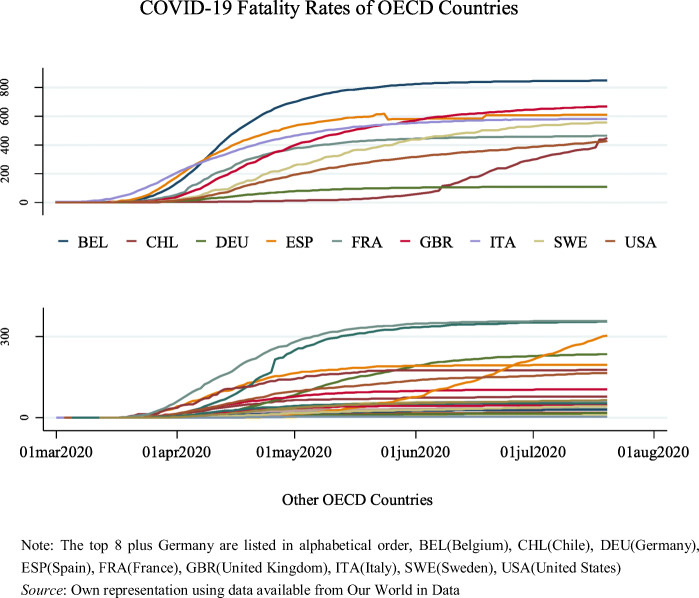
**Fatality Rates in OECD Countries (cumulated COVID-19 fatalities until 20 July 2020, per million population in 2018)**

**Fig. 2:**
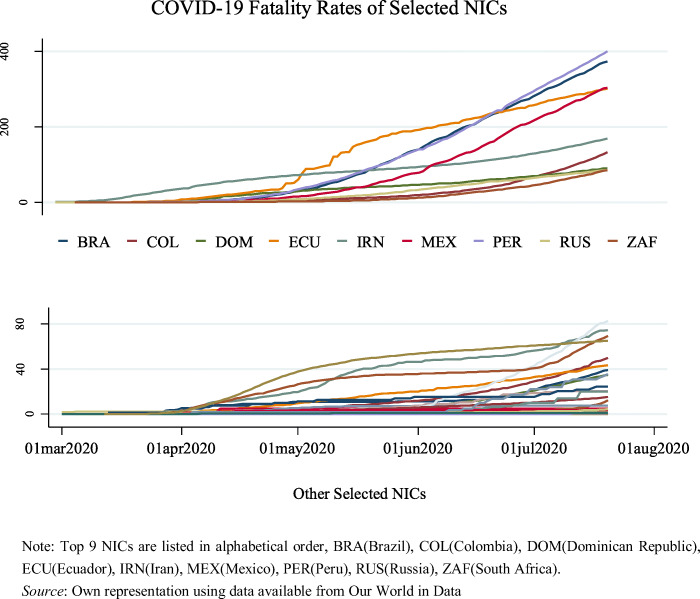
**Fatality Rates in Selected NICs (cumulated COVID-19 fatalities until 20 July 2020, per million population in 2018)**

Fatality rates (measured by deaths per million of population (population figures for 2018)) differ considerably across the OECD countries, see Fig. 1; in most OECD countries, the peak in terms of fatality rates had apparently been reached by the end of June, 2020. It is clear that Belgium is leading concerning the fatality rate among the OECD countries since April this year. Meanwhile, the death ratios of COVID-19 in the UK, France, and the US rise consistently. Although both Spain and Italy still have very high fatalities, the curves have become flatter in mid-2020. Looking at the growth trend from the graph and complementary statistics, countries of particular concern are Chile and Mexico, their death rates have soared since the beginning of June. Figure 2 below shows that the majority of the selected NICs generally have lower fatality ratios than OECD countries, but since late April 2020 experienced a rapid increase, especially in Peru, Brazil and Mexico; most of the countries in the top 9 list (NICs) are from South America.

The results for OECD countries indicate considerable differences in fatalities. In the subsequent ranking of countries (see Table 1) one can see that on the basis of fatality rates at late July 2020, the top five countries were Belgium, the UK, Spain, Italy and Sweden, followed by France, Chile, and the US. The five best performing countries were (in descending order) Japan, South Korea, the Slovak Republic, Australia, and New Zealand. Among the big economies with a rather favorable record in Europe – and with high levels of international trade and tourism linkages, including with China – is Germany, ranked 17, whose fatality ratio was around 1/4 of that of the US (two months prior the ratio was ½). Three ranking places behind Germany are Denmark, Austria and Turkey; the latter’s fatality ratio is only about 1/13 of that of Belgium. Press reports (see, e.g., Beisel 2020) have argued that Belgium’s death rate is particularly high since care homes with one COVID-19 fatality will record all subsequent mortality cases – without testing – as being linked to COVID-19. We also listed the ranking of a group of selected NICs in Table 2. Peru, Brazil, Mexico, and Ecuador are the top 4, and these countries all have above 300 fatalities per million population, the rate of deadly cases in Ecuador is almost twice that of the following country Iran. Brazil, as a big country in Latin America, and Iran, as a big country (in terms of population figure) in Asia, have fatality ratios that are slightly below that of the United States and roughly the same as Portugal, respectively. Russia is close to the fatality ratio of Austria. China, where the novel coronavirus emerged, kept the fatality rate below 5 per million. In any case, it is remarkable that countries show considerable differences in terms of fatality rates. Given the fact that hospitals and acute care beds, respectively, are less available in NICs than in OECD countries, and one may assume that NICs might have some underreporting of fatality ratios, but so far there is no evidence from comparative research on this issue.

**Table 1:** COVID-19 Fatality rates in OECD countries (cumulated COVID-19 fatalities until 20 July 2020, per million population in 2018)

Rank	Country	Fatalities per mn	Rank	Country	Fatalities per mn	Rank	Country	Fatalities per mn
1	Belgium	845.59	14	Luxembourg	177.32	27	Poland	42.91
2	United Kingdom	667.3	15	Portugal	165.64	28	Czech Republic	33.52
3	Spain	607.85	16	Colombia	132.38	29	Lithuania	29.39
4	Italy	579.62	17	Germany	108.45	30	Iceland	29.3
5	Sweden	556.38	18	Denmark	105.49	31	Greece	18.61
6	France	461.93	19	Austria	78.94	32	Latvia	16.44
7	Chile	444.81	20	Turkey	65.11	33	Japan	7.79
8	United States	424.57	21	Hungary	61.7	34	South Korea	5.77
9	Netherlands	357.69	22	Finland	59.2	35	Slovakia	5.13
10	Ireland	355.02	23	Slovenia	53.39	36	Australia	4.78
11	Mexico	303.91	24	Estonia	52.02	37	New Zealand	4.56
12	Canada	234.54	25	Israel	47.25			

*Source*: Own representation using data available from Our World in Data

**Table 2:** COVID-19 Fatality rates in selected NICs countries (cumulated COVID-19 fatalities until 20 July 2020, per million population in 2018)

Rank	Country	Fatalities per mn	Rank	Country	Fatalities per mn	Rank	Country	Fatalities per mn
1	Peru	399.95	13	Turkey	65.11	25	Belize	5.03
2	Brazil	373.96	14	Argentina	49.70	26	Paraguay	4.35
3	Mexico	303.91	15	Bulgaria	43.18	27	Malaysia	3.80
4	Ecuador	301.14	16	Albania	38.92	28	Georgia	3.76
5	Iran	168.92	17	Suriname	35.80	29	Jamaica	3.38
6	Colombia	132.38	18	Azerbaijan	34.91	30	China	3.23
7	Dominican Republic	90.43	19	Guyana	24.16	31	Namibia	1.18
8	South Africa	84.86	20	Gabon	20.67	32	Jordan	1.08
9	Russia	84.57	21	Kazakhstan	19.97	33	Thailand	0.83
10	Guatemala	82.89	22	Indonesia	15.15	34	Botswana	0.43
11	Bosnia and Herzegovina	74.68	23	Costa Rica	12.17	35	Fiji	0.00
12	Serbia	69.37	24	Cuba	7.68			

*Source*: Own representation using data available from Our World in Data

Certain EU countries with very high fatality ratios have at some point suffered critical situations in terms of acute care capacities in hospitals as is witnessed by the relocation of COVID-19 hospital patients from Italy and France to Germany. Among the countries covered in the graph and the table above, Sweden, with its rather liberal epidemic policy – with limited lockdowns imposed on Swedish families early on – does not show a favorable performance in the field of COVID-19 fatalities; Sweden, the Netherlands and the UK are three countries which placed an early emphasis on herd immunity. One cannot easily argue that countries with high fatality rates have been strict in early lockdown measures and shutdowns, respectively. Among the countries with rather low fatality rates, Greece is remarkable as a country which imposed strict regulatory quarantine measures rather early on. A systemic approach requires a broad econometric analytical approach.

Based on our assessment of fatality and excess fatality rates in OECD countries, we choose to use COVID-19 death rates in the empirical part, holding the COVID-19 case rates constant. This reflects the heterogeneous standards of measurement in the different countries as well as the random spread of the pandemic between the countries. In principle, to have the infection rates instead of the case rates would be preferable but these figures are unfortunately biased and unreliable. It turns out that the most important predictor for the number of deaths is the number of cases. Hence, when attempting to estimate the impacts that health and environmental variables have on the number of deaths, we therefore include the number of cases in order to avoid omitted variable bias and improve the precision of our results.

## Empirical model and data

### Deaths

Explaining epidemic fatalities is a rather difficult challenge – certainly with a limited sample of data. Among the key variables to be considered are predispositions in the various OECD countries’ populations and possibly influences relevant to the respiratory system. This potentially includes, for example, air quality aspects and thus crucial environmental aspects.

The choice of the dependent variable is not straightforward. While one is typically interested in the infection fatality rate, i.e. the ratio of deaths to infections, this number is unreliable, especially in an ongoing pandemic. This is due to the difficulty in accurately estimating the number of infections in a cross-country perspective, as different countries have varying testing regimes. Here, we choose to focus on the death rate per million as there is less variation in how deaths from COVID-19 are tested and reported across countries. However, as different countries were affected to differing degrees by the virus due to a combination of luck and successful policies, the death rate per million is not necessarily informative on its own. To get around this issue, we include the reported number of cases by country in all our regressions, as keeping the number of cases constant allows for a more informative comparison of the factors that affect deaths per million. Finally, we include an alternative specification using the average growth rate of the total deaths across the sample period. We are aware that even the measurement of cases involves some differences between countries, which leads us to interpret our results with caution.

Our main independent variable of interest is pollution, specifically PM2.5. Our first choice for this variable is exposure in the largest functional urban area (FUA). In most countries, the virus hit large cities the hardest so most victims of the virus would be living in the largest city or in cities that are very similar in terms of air quality. However, this variable is not available for the all countries. Five OECD countries are missing this variable, along with the extended sample of non-OECD countries. The alternative variables are mean exposure to PM2.5 for the whole country, and exposure in the largest *available* city. We recognize that there may be endogeneity associated with the use of pollution, in particular there might be confounding variables in terms of population density and economic activity. We have not found a convincing instrument that works on a cross-country level, so we cannot claim that our results are causal. However, we deal with issues of omitted variable bias by including a selection of control variables described below. However, our results may be reporting correlations rather than causal relationships, and should be interpreted with caution.

To control for other potential factors affecting the lungs, we also include the percentage of smokers in each country. A priori it is not clear what effect we should expect from this variable, as smoking has also been linked to lower case fatality of COVID-19.

More recent insights from corona fatalities show that fatality rates are higher for the elderly, and that COVID-19 attacks the blood circulatory system and related cells in addition to the respiratory system. Being overweight has also been suggested as a risk factor in COVID-19. The health condition of the population at large thus appears to be an important factor, and we therefore control for the population aged 65 and above in the largest FUA or median age, the percentage share of the population that is overweight, the death rate from cardiovascular disease and the prevalence of diabetes.

Furthermore, predisposition factors in the health system could play a role. There could be weak points in the availability of adequate personal protective equipment for medical personnel and care personnel in nursing homes. We therefore include the number of hospital beds per 1000 as a proxy for the quality of the health care system in the extended sample due to a high correlation with the overweight variable in OECD countries. In robustness checks (see in Appendix Tables 11 and 12), we also used findings of the Global Health Security (GHS) Index in both samples, but find less convincing results. We also include the percentage of smokers in each country to control for other potential factors affecting lung capacity and health, thus leading to a higher risk of respiratory infections. However, a priori it is not clear what effect we should expect from this variable, as smoking has also been linked to lower case fatality of COVID-19, and according to a review report from WHO until end of June, the impact from smoking is contradicted in existing studies (2020g).

Policy response is an important factor in determining the impact of the virus. However, high fatality rates more or less force the government to adopt strict shutdown and lockdown measures, since otherwise the intensive care capacities in hospitals would quickly be overwhelmed. This risk always exists once the so-called R infection factor exceeds unity (R indicates a critical parameter of the spreading function of the virus). With R > 1, the system moves to an exponential virus diffusion function as one infected person will infect more than one other person so that it is only a question of time until hospital capacities are exceeded. When successful, such measures reduce the transmission rates and the number of cases which, in turn, lowers the death rate per million. While most of the policy response would be captured in the number of cases, we do include variables to control for policy responses. In the main regression, we include a dummy for whether a country adopted a herd immunity policy at the start of the pandemic, with two alternative variables: the mean value of the policy stringency response as reported by Our World in Data (OWID), as well as the speed of the policy response as measured by the number of days between the date the first 10 cases were reported and the date on which the country implemented travel restrictions.

As there is an important element of learning over time from the virus, we assume that countries hit by the crisis earlier might see higher deaths than those affected later. We therefore include a variable indicating the number of days from January 1st until the first case was recorded in a country (for some countries this number is negative).

### Cases

As particulate matter can facilitate the spread of a virus (there are links between PM2.5 and precipitation as well as other meteorological variables – see, e.g., the study on New York by Adhikari and Yin 2020) we also look at whether pollution has an effect on the number of cases per million, and the average growth rate of the total cases during the sample period. In determining the number of cases, economic, demographic and policy responses are likely more important than health factors. We therefore control for more country specific issues than when looking at the number of deaths. We include GDP per capita to control for income level. While richer countries may generally be better equipped to tackle a pandemic, COVID-19 has hit rich countries earlier. Furthermore, richer countries tend to have higher mobility, which can facilitate the spread of the virus. We therefore expect a positive sign on GDP per capita. As the virus will spread faster in more densely populated areas, we include population density. As policy response is key in containing the spread of the virus, we include the herd immunity dummy as well as the speed of the policy response.

Finally, we include selected health variables. We reason that a less healthy population will likely have a higher number of more severe cases, thus recording a larger fraction of the cases. For the OECD sample, due to the low number of observations, we only include the fraction of smokers and of overweight people. In the full sample we add the cardiovascular disease (CVD) death rate and the diabetes prevalence, as well as the timing of the outbreak of the pandemic in each country.

### Sample

To facilitate comparison across countries, we use World Bank classifications to exclude countries defined as low income and as fragile/in conflict. In robustness checks, we consider the subsamples of high and upper middle income countries, as well as OECD only. The pandemic hit OECD and higher income countries earlier, so these countries may be further along the infection curve. A list of countries included in each sample can be found in the Appendix in Table 10.

The variables are described in the following Table 3, with summary statistics in Table 4. A correlation matrix is included in the Appendix (Table 9).

**Table 3:** Description of the Variables

Variables	Description	Source	Expected sign	Time period
Deaths per million	Total deaths attributed to COVID-19 per million people	OWID		31.12.2019–20.07.2020
Cases per million	Total confirmed cases of COVID-19 per million people	OWID	+	31.12.2019–20.07.2020
Avg. growth rate, deaths	Average growth rate of COVID-19 deaths	OWID		31.12.2019–20.07.2020
Avg. growth rate, cases	Average growth rate of COVID-19 cases	OWID		31.12.2019–20.07.2020
PM2.5 in largest FUA	Mean exposure to PM2.5 in the largest functional urban area	WHO	+	2017
PM 2.5 Exposure	Mean annual exposure to PM2.5 (micrograms per cubic meter)	World Bank	+	2017
PM2.5 in most populous city	PM2.5 exposure in the most populous city available	WHO	+	2017
Median age	The median age of the population, UN projection for 2020	OWID	+	Latest year available
Percent over 65, largest FUA	The share of the population aged 65 and above in the largest functional urban area	WHO	+	2017
Percent overweight	Estimated share of the population that is overweight	WHO	+	2016
Percent smokers	The average percentage of male and female smokers	OWID	±	Latest year available
Diabetes prevalence	Diabetes prevalence (% of population aged 20 to 79)	OWID	+	2017
CVD Death rate	The death rate from cardiovascular disease	OWID	+	2017
Hospital beds/ thousand	Hospital beds per 1000 people	OWID	–	Latest year available
GHS Index	The overall score of the Global Health Security Index (0–100, 100 = highest score)	GHS Index 2019	_	2019
Herd immunity policy	A dummy variable equal to 1 if a country applies the herd immunity policy (UK, Sweden, Netherlands, and Brazil)	News items*	+	2020
Days until first case	The number of days from 1st January until the first case was recorder, own calculation	OWID	_	01.01.2020–20.07.2020
Days until intl. Travel control	Days from first 10 cases until any international travel controls issued, own calculation	OxCGRT	_	01.01.2020–20.07.2020
Mean Policy stringency	Mean of stringency index from the first date data available to 20th July 2020	OxCGRT	_	01.01.2020–20.07.2020
GDP per capita	Gross domestic product at purchasing power parity (constant 2011 international dollars)	OWID	+	Latest year available
Population density	Number of people divided by land area, measured in square kilometers	OWID	+	Latest year available

OWID uses the data from the European Centre for Disease Prevention and Control (ECDC), OxCGRT represents the Oxford Covid-19 Government Response Tracker (Hale et al. 2020). Covariates included in the OWID dataset are from several sources, see: https://github.com/owid/covid-19-data/blob/master/public/data/owid-covid-data-codebook.md

*For the Netherlands, see the speech by Prime Minister Mark Rutte on March 16, 2020: https://www.government.nl/documents/speeches/2020/03/16/television-address-by-prime-minister-mark-rutte-of-the-netherlands; for Sweden, see public comments from the country’s chief epidemiologist Anders Tegnell https://www.svd.se/tegnell-flockimmunitet-inte-huvudtaktiken?fbclid=IwAR0ESWZX8S_QbSWcnSCKGaHxhnw_gBxTxn88CsHwoAWOMlCB7i1BhDTIPPI; for the United Kingdom, comments from the United Kingdom’s Chief Scientific Adviser Sir Patrick Vallance: https://www.ft.com/content/38a81588-6508-11ea-b3f3-fe4680ea68b5. The article from Bhatt and Parikh on the website of the abcNews news network (https://abcnews.go.com/US/vaccine-reach-herd-immunity-scientists/story?id=71662733) provides information about herd immunity policy in Brazil, the Netherlands, and Sweden (2020)

*Source*: Own representation

**Table 4:** Summary Statistics

	N	Mean	St.Dev	min	max
Deaths per million	132	102.5	188.37	0	1237.55
Cases per million	132	3156.73	4967.14	2.61	37,016.93
Average growth rate of deaths	122	5.71	3.03	0	25
Average growth rate of cases	132	8.08	2.67	1.91	16
PM2.5 in largest FUA	32	13.59	5.63	5.8	25.3
PM2.5 air pollution, mean annual exposure	147	23.95	17.76	5.86	99.73
PM2.5 in most populous city available	84	25.33	19.03	5	92
Percent over 65, largest FUA	32	16.46	3.32	7.93	23.86
Median age	127	33.58	8.11	16.8	48.2
Percent overweight	143	52.11	15.76	18.3	87.9
Percent smokers	109	22.28	8.89	4	43.65
Diabetes prevalence	131	8.11	3.98	.99	22.02
Death rate from CVD	129	236.11	119.08	79.37	724.42
Hospital beds/thousand	118	3.29	2.34	.3	13.05
GHS index	139	44.95	14.02	16.2	83.5
Days from Jan 1 until first case	131	60.62	19.62	13	136
Herd immunity policy	136	.03	.17	0	1
Days until travel restrictions, from 10 cases	129	−18.59	37.36	−118	147
Mean policy stringency	135	46.09	9.26	8.06	67.02
GDP per capita	127	24,278.45	21,031.96	2064.24	116,935.6
Population density	132	297.46	973.63	.14	7915.73
Observations	132				

Overall, we believe our choice of sample allows us to make informative observations, and that our variables cover important sources of omitted variable bias. However, the small sample size and the cross-sectional nature of the study leaves room for future researchers to expand on our methodology. Once the pandemic has run its course, researchers will be able to extend the sample size and adopt more complex methodologies, thus allowing for more conclusive evidence on the causality of the relationships documented in this paper.

## Empirical results

### OECD countries

#### Deaths

The results of our empirical analyses for OECD countries are reported in Table 5, where a range of regression equations explaining fatality rates are considered. In column (1), we include health variables, while in column (2) we add policy variables. In column (3), we show alternative policy variables. In columns (4) and (5) we report alternative specifications: column (4) indicates the results of using the mean pollution exposure variable, thus extending our sample to 37, while column (5) reports the results when the growth rate of the deaths is the outcome variable.

**Table 5:** OECD, COVID-19 Deaths

	(1)	(2)	(3)	(4)	(5)
	Deaths per million	Deaths per million	Deaths per million	Deaths per million	Avg. growth rate, deaths
Variables	OECD	OECD	OECD	OECD	OECD
PM2.5 in largest FUA	10.46*	11.43**	6.38		0.02
	(5.17)	(4.54)	(5.72)		(0.03)
PM2.5 air pollution, mean annual exposure				12.16*	
				(7.03)	
Cases per million	0.03**	0.03**	0.03**	0.03**	
	(0.01)	(0.01)	(0.01)	(0.01)	
Percent over 65, largest FUA	5.10	0.97	2.73	3.29	0.01
	(7.29)	(7.94)	(7.70)	(9.08)	(0.06)
Percent overweight	10.44***	11.19***	7.29	11.44**	0.12***
	(3.16)	(3.42)	(4.35)	(4.13)	(0.03)
Percent smokers	−5.46	−1.41	−1.45	−1.41	0.06
	(6.49)	(5.77)	(5.28)	(6.48)	(0.06)
Death rate from CVD	−1.15**	−0.73*	−0.41	−0.74*	−0.01***
	(0.49)	(0.36)	(0.37)	(0.37)	(0.00)
Diabetes prevalence	−31.41*	−22.76	−19.75	−24.11	0.38***
	(17.54)	(19.41)	(18.48)	(22.21)	(0.13)
Days from Jan 1 until first case		−4.02**	−3.76**	−4.14**	−0.04**
		(1.76)	(1.67)	(1.85)	(0.02)
Herd immunity policy		229.97***	255.21***	224.83**	2.20***
		(78.34)	(86.89)	(85.82)	(0.67)
Days until travel restrictions, from 10 cases		−0.55		−0.61	
		(0.85)		(0.90)	
Mean policy stringency			8.81**		
			(3.94)		
Constant	−226.23	−263.59	−442.33*	−303.28	−1.38
	(222.57)	(234.97)	(226.43)	(250.76)	(2.38)
Observations	32	32	32	32	32
R-squared	0.56	0.69	0.71	0.68	0.69

Robust standard errors in parentheses

*** *p* < 0.01, ** *p* < 0.05, * *p* < 0.1

The effect of pollution is positive and significant at the 10% level or below in each regression, except for the growth rate of deaths and use the mean policy stringency in model 3. However, due to the small sample size, it is not surprising that the precision of the results is somewhat variable. The results indicate that an increase of 1 μg/m3 PM2.5 in the mean exposure to PM2.5 in the largest city is associated with an increase in deaths per million of around 10. However, while our results generally show that an increase in PM2.5 concentration appears to be associated with a higher number of deaths from COVID-19, the results are not highly robust to alternative specifications (not reported). Further research on a larger sample, ideally with a higher spatial resolution, is also required before one can conclude that an increase in pollution *causes* a higher fatality rate from COVID-19. However, our regressions do indicate that higher pollution is associated with more COVID-19 fatalities.

In terms of the controls, we find that total cases per million are associated with a higher number of deaths per million, which is not surprising (Table 5). One more case is associated with around 0.03 more deaths per million in a country. A large elderly population in the largest city is associated with a higher number of deaths, but the results are not statistically significant. The overweight variable is also statistically significant except for specification (3), and it has the expected positive sign. The sign of the percent of smokers is inconsistent and statistically insignificant. The death rate from CVD has an unexpected negative sign, statistically significant in all regressions with the exception of specification (3). While we cannot say with certainty why this is, there are several potential explanations: e.g., countries with a high number of CVD deaths in the past may have more Acute Intensive Care units, or higher CVD death rates means that there are fewer at-risk individuals left in the population. Diabetes prevalence also appears insignificant, except in columns (1) and (5), where the results seem to indicate that a higher prevalence of diabetes is associated with a higher growth rate of the deaths. The number of hospital beds also appears to be insignificant. Note that the small sample size works against the precision of these results, and thus they should not be taken as clear evidence that none of these variables affect COVID-19 death rates.

Of the policy variables, the herd immunity policy variable appears the strongest: it is statistically significant, and the coefficient is large. A country that initially pursued a policy of herd immunity appears to have around 225–255 more deaths per million than other countries, as well as a 2.2 percentage points higher average growth rate. Travel restrictions appear not to significantly affect the number of deaths. In column (3), we introduce the mean policy stringency variable as a control, while dropping the herd immunity policy control. Policy stringency appears to be statistically significant. There is one potential caveat: the stringency of policy could be endogenous to both the severity of the outbreak and the fatality rate: A harder hit country might introduce very strict regulations once the true nature of the threat has been acknowledged. One may emphasize that the OECD should provide full data coverage for all countries – with only 32 of the 37 OECD countries giving the relevant data there is a critical lack of data. Moreover, the OECD would be wise to contribute to collecting excess fatality data for all its member countries in a timely manner.

#### Cases

We explore the relationship between pollution and the number of cases for OECD countries in Table 6. In column (1), we include basic country-level characteristics of GDP (per capita GDP, purchasing power parity) and population density; it is not surprising to see that per capita income has a positive impact on infection numbers (see column (1) in the table for cases in OECD countries) since a higher income goes along with more international trade contacts and international tourism contacts which in turn typically raise the probability of internationally transmitted epidemic infection. In column (2), we introduce policy variables, and in column (3), we add the health controls. Column (5) reports the results from using the alternative PM2.5 variable on the full OECD sample, and column (6) shows results from looking at the average growth rate of the cases.

**Table 6:** OECD, COVID-19 Cases

	(1)	(2)	(3)	(4)	(5)
	Cases per million	Cases per million	Cases per million	Cases per million	Avg. growth rate, cases
Variables	OECD	OECD	OECD	OECD	OECD
PM2.5 in largest FUA	194.2	286.4	217.3		0.063
	(241.8)	(266.2)	(201.7)		(0.0836)
PM2.5 air pollution, mean annual exposure				107.5	
				(95.43)	
GDP per capita	0.107***	0.089	0.095	0.044	3.24e-05
	(0.037)	(0.056)	(0.0714)	(0.041)	(2.57e-05)
Population density	−7.404	−12.71	−8.015	−3.641	0.008
	(6.989)	(8.730)	(8.581)	(7.138)	(0.007)
Days from Jan 1 until first case		−39.98	−55.70	−33.78	0.001
		(34.36)	(43.15)	(36.16)	(0.017)
Herd immunity policy		2736	2280	618.1	−0.806
		(1955)	(2667)	(2004)	(1.972)
Days until travel restrictions, from 10 cases		25.91	12.61	25.96	−0.008
		(19.96)	(23.14)	(20.56)	(0.012)
Percent over 65, largest FUA			−265.4		−0.067
			(296.4)		(0.088)
Percent overweight			75.93	106.0	0.143**
			(86.14)	(68.88)	(0.064)
Percent smokers			123.8	5.875	
			(162.1)	(105.6)	
Constant	−2543	−846.4	−3467	−4341	−2.437
	(3281)	(4282)	(5569)	(3691)	(3.753)
Observations	32	32	32	37	32
R-squared	0.139	0.256	0.339	0.195	0.364

Robust standard errors in parentheses

*** p < 0.01, ** p < 0.05, * p < 0.1

The coefficient of the pollution variable shows a large positive effect of pollution, however the results are not statistically significant in any specification. Indeed, while the variables generally have the expected sign, most of the variables in Table 6 are not statistically significant. As the small sample size does not allow us to say with any certainty whether the lack of strong results is due to the small sample or due to the relationships between the variables being non-existent, we increase the sample size by adding other middle and high income countries.

### Middle and high-income countries

#### Deaths

We report the results for the regression on cases in Table 7 In the first column, we report the results when controlling for population health, while in column (2) we include country characteristics and policy variables and include regional dummies. In column (3), we use the alternative pollution variable, pollution in the largest city, and in column (4) we restrict the sample to richer countries, column (5) removes lower middle income countries.

**Table 7:** Full sample, COVID-19 Deaths

Variables	(1)	(2)	(3)	(4)	(5)
	Deaths per million	Deaths per million	Deaths per million	Deaths per million	Avg. growth rate, deaths
	full sample	full sample	full sample	U. middle & High income	full sample
PM2.5 air pollution, mean annual exposure	−0.915	−0.800		−1.607	0.021*
	(0.716)	(0.770)		(1.786)	(0.012)
PM2.5 in most populous city available			0.697		
			(0.879)		
Cases per million	0.010**	0.008**	0.0147***	0.008*	
	(0.005)	(0.004)	(0.005)	(0.004)	
Median age	1.719	−4.887	−6.732	−4.466	−0.116*
	(2.292)	(4.368)	(5.885)	(4.926)	(0.059)
Percent overweight	2.127**	1.973	4.564*	2.744	0.065***
	(0.937)	(1.376)	(2.333)	(2.261)	(0.017)
Percent smokers	0.112	1.771	1.361	1.898	0.034
	(1.675)	(1.719)	(2.208)	(2.376)	(0.030)
Death rate from CVD	−0.257**	−0.186	−0.146	−0.329**	−0.001
	(0.104)	(0.117)	(0.150)	(0.155)	(0.002)
Diabetes prevalence	−9.840**	−3.510	−8.876	0.267	−0.110**
	(3.808)	(3.569)	(7.402)	(4.489)	(0.055)
Hospital beds/thousand		−6.113	−7.030	−8.458	0.097
		(7.910)	(9.519)	(8.985)	(0.117)
Herd immunity policy		230.3***	188.6***	222.7***	3.310***
		(56.77)	(59.36)	(63.85)	(0.948)
Days until travel restrictions, from 10 cases		0.539	0.521		
		(0.384)	(0.521)		
Days from Jan 1 until first case		−3.082**	−4.767**	−4.641***	−0.0467***
		(1.193)	(1.890)	(1.596)	(0.013)
Constant	55.29	299.8**	320.6*	301.2	8.369***
	(56.70)	(139.6)	(191.7)	(193.0)	(1.590)
Observations	108	104	75	77	98
R-squared	0.337	0.576	0.627	0.586	0.336
Region dummies	NO	YES	YES	YES	NO

Robust standard errors in parentheses

*** p < 0.01, ** p < 0.05, * p < 0.1

The results of the deaths are ambiguous. In most specifications, we cannot detect a significant relationship, and the coefficient is negative. Using the alternative PM2.5 variable, while still not statistically significant, does show a positive association with deaths. Finally, the growth rate of deaths appears to have a positive correlation with the PM2.5. The results thus do not strongly support the results from the OECD counties. A potential explanation for the significant sign of PM2.5 in OECD countries could be that the life expectancy in OECD countries is higher than in middle-income countries – if there is a kind of long-term “fatigue effect” on the respiratory system, PM2.5 could indeed play a critical role in OECD countries here.

Unsurprisingly, the number of cases has a positive and significant effect on the number of deaths across all specifications. The point estimate indicates that increasing the cases per million by 1 increases the number of deaths by about 0.01.

The effects of the health variables are somewhat surprising. Neither the share of smokers nor the median age appears to have a significant impact on deaths. Median age appears to have a negative effect on the average growth rate of deaths. Both prevalence of cardiovascular disease (CVD) and diabetes appear with a negative sign when significant. However, neither result is consistent across specifications. Percent of overweight people appears to increase the deaths per million, although this variable is only significant in some specifications.

Preparedness of the health care system measured as beds per 1000, appears to lower the number of deaths by about 10 per extra bed, but the results are not statistically significant. We also used GHS indices as alternative measurements of the quality of the health care system. The results are not shown, but the coefficients for the overall GHS score as well as relevant sub-indices were negative and statistically insignificant. The herd policy variable appears to be important as it is statistically significant in all regression results, and the coefficient is large. A country that initially pursued a policy of herd immunity seems to have between 190 and 250 more deaths per million than other countries, and a 3 percentage points higher growth in deaths.

Finally, the date of the first case appears quite important: pushing back the arrival of the virus by just one day – or a week - is associated with fewer deaths per million. The implication is that effective WHO communication strategies of early warning systems about the new virus were of crucial importance as were national policy measures to slow down the spreading of the virus nationally and internationally in the first half of 2020.

Overall, our empirical results point to a tentative conclusion that there may be an adverse effect of pollution and exposure to PM2.5 on the severity of the COVID-19 pandemic. However, the results presented above are subject to several limitations, and should be interpreted with caution. Due to data constraints in an ongoing pandemic, we rely on cross-sectional variation, thus we are unable to establish the causality of the relationships. Further, there are likely measurement errors in both the number of cases and deaths, and we do not know whether these are random or if they introduce significant bias in our results. Future research should take care to establish causality and use alternative and updated numbers for the deaths and the cases – including using excess mortality rather than the reported mortality. Still, we continue below with the policy implications of the results, noting that further research is necessary for stronger conclusions to be drawn.

#### Cases

We report the results for the regression on cases in Table 8. In the first column, we report the results when controlling for population health, while in column (2), we include country characteristics and policy variables and include regional dummies. In column (3), we use the alternative pollution variable, pollution in the largest city, and in column (4), we restrict the sample to richer countries, column (5) removes lower middle income countries.

**Table 8:** Full sample, COVID-19 Cases

Variables	(1)	(2)	(3)	(4)	(5)
	Cases per million	Cases per million	Cases per million	Cases per million	Avg. growth rate, cases
	full sample	full sample	full sample	U. middle & High income	full sample
PM2.5 air pollution, mean annual exposure	93.91*	82.87*		153.5*	0.048***
	(52.02)	(47.26)		(85.59)	(0.014)
PM2.5 in most populous city available			43.42*		
			(25.80)		
Median age of the population	−66.74	−130.1*	−267.3***	−152.3	−0.05
	(78.03)	(72.35)	(95.44)	(95.68)	(0.046)
Percent overweight	143.1***	−15.34	120.6***	35.67	0.065***
	(38.84)	(38.93)	(36.61)	(46.06)	(0.020)
Percent smokers	−35.95	57.63	93.84	96.63	−0.003
	(73.88)	(80.18)	(94.29)	(106.4)	(0.035)
Death rate from CVD	−11.00***	−0.727	−10.01	−0.647	−0.003
	(4.034)	(4.554)	(6.266)	(5.895)	(0.002)
Diabetes prevalence	142.4	−4.058	298.4*	−90.38	−0.083
	(90.67)	(101.3)	(169.3)	(138.3)	(0.060)
GDP per capita		0.156***	0.0547*	0.144***	1.48e-05
		(0.056)	(0.029)	(0.050)	(1.23e-05)
Population density		0.154	0.733*	0.257	−5.32e-06
		(0.608)	(0.434)	(0.470)	(0.0002)
Herd immunity policy		3357*	2990	3656**	1.335
		(1742)	(1921)	(1723)	(0.939)
Days until travel restrictions, from 10 cases		−23.00*	0.454	−19.58	−0.004
		(12.52)	(10.50)	(12.35)	(0.006)
Days from Jan 1 until first case		−16.21	−28.24	−34.54	0.021
		(26.08)	(23.16)	(35.59)	(0.014)
Constant	−1961	−1168	2712	−2964	5.021***
	(2845)	(3377)	(2582)	(5775)	(1.673)
Observations	108	106	75	77	106
R-squared	0.361	0.599	0.517	0.683	0.285
Region dummies	NO	YES	NO	NO	NO

Robust standard errors in parentheses

*** p < 0.01, ** p < 0.05, * p < 0.1

The number of cases seems to increase with exposure to PM2.5. The coefficient is large and positive in all specifications, ranging from 40 to 150 more cases per million when mean exposure to PM2.5 is increased by 1 μg/m3 PM2.5. In absolute terms, this means that if Italy reduced its average pollution exposure to the level of Finland (from around 15 to around 5), the country could have seen roughly between 25 and 90,000 fewer cases. The results are statistically significant when controlling for the full set of covariates, as well as when restricting the sample to upper middle income and high income countries in columns and when looking at the average growth rate.

Interestingly, the median age of the population appears with a negative sign and statistically significant in the full regression. Here, increasing the age of the population by 1 year is associated with a fall in the number of cases by more than 130. It is possible that the higher mortality rate among older people makes them more likely to heed social distancing guidelines, thus reducing the total number of cases. A similar explanation could be posited for the negative effect of CVD deaths. However, when including economic and policy variables, the effect of CVD disappears.

The share of overweight people appears to increase the total number of cases while statistically significant. However, the effect disappears when including regional dummies, possibly due to the regional variation of obesity. The other health variables appear to not play a large role in the spread of the virus.

Of the country characteristics, we see that GDP has a significant positive effect on the number of cases. This is not surprising as the virus hit rich countries in Europe and North America first, and the effect may well disappear when the virus runs its course in the lower income countries. It should be considered that high per capita income countries tend to have both relatively strong international trade and investment relations (including) and rather elevated levels of international tourism activities – both with respect to international tourism abroad and with respect to incoming foreign tourists; hence a higher per capita income and higher virus spreading intensity will go together which explains the link between per capita income and infections.

The results of the policy variables are surprising. The herd immunity policy has a large coefficient – a country initially pursuing herd immunity has more than 3000 more cases per million than other countries, significant in columns (3) and (4). If these results were causal, the magnitude for a country like Sweden, with a population of 10 million and 80,000 cases, 30,000 cases (or 37.5%) might have been due to the initial herd immunity policy. It should be noted that the herd immunity finding is crucial not just in a medical perspective; higher infection numbers in herd immunity countries such as the UK, the Netherlands and Sweden could also bring about stronger output declines which in turn undermine economic recovery in these countries as well as trading partners due to potential spillover effects. However, while the countries that followed herd immunity at first appear somewhat arbitrary and thus potentially random, more research needs to be done to fully establish this as a causal relationship.

While the speed of any international travel controls appears to reduce the number of cases, the result is only statistically significant at the 10% level in the full sample with regional dummies. Results are similar for other policy variables (e.g., time until stay at home orders or the cancellation of public events), and are not reported. Similarly, we find no effect of the speed at which the virus first appeared in a country on the total number of cases.

As regards the results for the full sample in Table 8, one should consider: In all four of the models (1) to (4), air quality problems are significant drivers of infections; in two of these four equations, obesity and the herd immunity variable show a significant impact. In three of the four equations, the per capita income variable is significant for higher infections (recall that per capita income, based on PPP figures, can be considered to be a proxy for international contact intensity). Overweight is also significant in column (5) for the growth rate of infections. As regards the obesity variables in high income countries and low income countries, one should keep in mind that in relatively poor countries, being underweight is rather the more pressing problem in terms of health system challenges and as per capita income and the weight of both children and adults are rising, health care expenditures are also rising (Bansal and Zilberman 2020) so that one should avoid a simplistic interpretation of the obesity variable in the various models. The more low income countries are included, the more important problems related to being underweight – again, in relatively poor countries a frequent problem – will be part of a broad full sample group: In both Tables 7 and 8 – the former referring to COVID-19 fatality ratios – the obesity variable is significant in the regression in column (3) for the rather small group of 75 countries (higher and middle income group) while the overweight variable is no longer significant in the enlarged group of 104 countries of column (2) in Table 7 and in the group of 106 countries group of column (2) in Table 8.

Looking at the main fatality results for the full sample in Table 7, the cases are significant in all equations and the herd immunity variable always has a positive significant effect on fatalities, while postponing the international diffusion of the corona virus (variable: days from Jan. 1 until first case) always has a negative impact.

## Policy conclusions and research perspectives

The economic logic of the above empirical findings is that an epidemic policy of countries which slows down the international diffusion of the virus brings high benefits in terms of lower fatalities as well as in terms of economic welfare gains. Policy measures which slow down the pandemic are crucial as one can expect both a positive effect in terms of saving lives and a positive welfare effect from lower output losses and avoiding extra health care expenditures. Based on the sample of 104 countries in Table 7, a one (or two) week slowing down of the global epidemic would save 183,624 (or 367,249) lives if one assumes that the parameter estimated also applies to the world economy (3.082 cases/1 million * 7days *7.8 billion world population = 168,277.2, for country details see Table 13 in the Appendix); the 104 countries cover 79% of global output (the global death toll was reported to be 686,703 on August 3, 2020 (WHO 2020f)). Without entering a debate on the economic value of human life, it is clear that changes in fatality figures are politically relevant and the massive public investment in vaccination programs in OECD/G20 countries clearly reflect the political sensitivity of COVID-19 fatality numbers. At the same time, it is rather remarkable how low-profile the herd immunity policy debate in Sweden, the Netherlands, the UK and Brazil has been in the first half of 2020 – given the clear finding that a herd immunity policy raises the fatality ratios as shown in the empirical section.

To the extent that national epidemic policy measures help to slow down the international spread of the virus, and thus help reducing, for example, extra health care expenditures (related inter alia to testing and covering the hospital stays of COVID-19 patients) but also bring about output losses (e.g., from quarantine measures in the respective country), there is a certain economic trade-off of anti-pandemic policy measures. The only exception would be national vaccination programs as such programs would give protection directly to the persons vaccinated and indirectly lead to a reduced probability of non-vaccinated people in the respective country and its major trading partners (where trading includes, of course, international tourism). To the extent that neighboring countries with early vaccination programs generate relatively strong positive external effects, for example for adjacent countries, it might be possible to obtain an effective level of herd immunity via vaccination for both countries combined even if one of the countries considered has a critical share of people resisting a vaccination; in a post-vaccination scenario, the relatively high share of foreign tourists from France or Italy, for example, in Germany could contribute to herd immunity in Germany if the share of people vaccinated in France or Italy exceeds that of people living in Germany.

For the coronavirus pandemic, and indeed similar future epidemics, one can draw the conclusion that policy measures aimed at curbing the number of overweight persons in the population should be a key element of a consistent strategy aiming to reduce fatality ratios; moreover, air quality problems have also been identified as crucial problems so that sustainability policy could be considered to be a critical element of epidemic policy as well. As policymakers in the EU have emphasized the need to combine economic recovery programs in the corona shock year of 2020 and in the following years with a particular emphasis on enhanced climate policy and sustainability policy, respectively – see the results from the EU summit in Brussels in July 2020 (European Council 2020) – one may argue that part of such measures could be expected to reduce PM 2.5 air quality problems and thus should help people to live with the coronavirus more easily in the future; a more explicit emphasis on reducing air quality problems, however, seems to be adequate in Europe, the UK, the US and Newly Industrialized Countries in the future. Fighting obesity could have become an immediate new policy priority in EU/OECD/G20 countries once the first empirical results for COVID-19 fatalities in OECD countries had been published (Bretschger et al. 2020). One could still could start an OECD-wide or G-20 anti-obesity program – beyond the soft standard WHO projects - in late 2020, and indeed within a few months some successful intermediate targets could be achieved, thereby reducing global fatality rates. Such a program would generate extra benefits in many high income countries to the extent that happiness indicators are typically negatively linked to obesity in general; obesity also reduces life expectancy in high income countries (Bansal and Zilberman 2020). There is some risk that in developing countries - where certain problems related to undernourishment already existed in 2019 - the corona shock-related economic crisis of 2020 has reinforced these existing underweight problems in this group of countries.

A lack of efficient international policy co-operation has been a problem in sustainability and climate protection policy – the mechanism of international inefficiency encountered here could have a partial mirror image in epidemic policy. Herd immunity policy cannot be recommended. Governments eager to avoid high fatality ratios should try to postpone the arrival and spreading of the coronavirus, respectively. As regards vaccination as a potential way to stop the pandemic, one should emphasize that vaccination has an element of a positive – national or international - external effect, namely that not only the person who accepts the risks of a vaccination and indeed gets vaccinated is protected but they also help to protect other people from falling ill with the coronavirus; this interesting aspect cannot be covered here, but it matters to the extent that adequate subsidization of R&D for a new vaccine and of vaccination, respectively, could help to stop the pandemic in the medium term and thus to make herd immunity approaches indeed obsolete.

To the extent that higher per capita income represents an internationally more open economy and society, one may argue – unsurprisingly – that economic prosperity might go along with a higher health risk. Without further research one should, however, not overemphasize this aspect since medical progress in the sense of developing new vaccinations or new pharmaceutical products (i.e. new chemical formulations which are more effective in fighting the pandemic disease) could also be facilitated by more international trade and investment linkages. One should also not rule out that higher per capita income is associated with more legal and illegal immigration – with immigrant workers often living in rather crowded housing conditions facing a high risk of infections and infections in the immigrant community will often spread amongst the larger community. In this context, one may point to one key observation regarding the Spanish Flu of 1918/19 (Spinney 2017, chapter 15): The fact that so many people died in some of the wealthiest quarters of Paris was a puzzle for scientists until they understood exactly who it was who was dying – the high income families in these quarters would typically employ domestic servants who often lived in a separate part of the house in crowded living conditions which facilitated the spread of the disease amongst the employed personnel (almost one quarter of the fatality cases were maids working for wealthy families). As regards COVID-19 infections and fatalities, more research on the social dimension is needed in the future.

The coronavirus pandemic raises key questions from a medical, economic and political perspective. If there is to be some international solidarity, the international community could decide to allocate particular help to those countries with a high number of fatalities per million. While the regression model looks at fatalities in OECD countries and selected NICs, the next steps in research will be to include more countries, if possible all UN countries; a necessary step for broad policy recommendations in the context of a global pandemic. The reflections presented are thus only part of a broader analytical effort which in the end should not overlook critical links between medical and economic dynamics in an international pandemic. Countries with both high infection rates and high numbers of COVID-19 deaths have obviously suffered particularly negative shocks in production, namely to the extent that there was an infection-related decline of production, the effective labor input has reduced, or that strict regulatory shutdowns and lockdowns were imposed by government that were designed to fight the epidemic but brought the side effect of a negative supply and a negative (aggregate) demand shock. Given the simple fact that fatalities differ so much across OECD countries and NICs, respectively, one may argue that our regression findings cover at least a critical part of the analysis. There may also be special aspects in the medical perspective that we as economists would want to cover only in a more interdisciplinary research context; international differences in health systems and hospital quality thus could play a role which is only indirectly covered here, namely in the number of infections registered in the various countries. With these caveats in mind, one may focus on preliminary policy conclusions.

There is a range of key policy conclusions one could draw as it was shown that the COVID-19 fatality rates of OECD countries depend on the number of coronavirus infected people, the share of overweight people in the population and the PM2.5 concentration (at a national level or in the respective biggest city; the latter variable is a proxy for air quality problems which have increased over decades in the major cities of OECD countries). As regards the air quality variable, further investigation is required in the future in order to clearly identify the relevance of air quality problems for infection dynamics and the fatality ratios in OECD countries and NICs, but one may also hope that more internationally comparative regional studies could be useful here.

As regards policy conclusions with respect to OECD countries and NICs, one may point out the following four key aspects:

- Leading OECD countries were rather strongly exposed to COVID-19 – at least as regards the fatality ratios. To the extent that strong trade, investment and tourism links with China have played a role in this aspect, effective epidemic policy in China is a prerequisite to restore international economic relations on a broad scale in a sustained manner. As regards cooperation between OECD countries and China in the field of health policy, the WHO still plays a crucial role - even as the US wants to leave the organization. The Trump Administration’s economic and health policy has been rather contradictory over years (Welfens 2019).
- Countries which are heavily dependent on tourism in both Europe and Asia could play a crucial role in new outbreaks as international tourism is typically associated with often crowded locations in urban areas, such as restaurants, bars, discos etc. as well as beaches and other leisure areas. The nature of the pandemic makes it crucial that OECD countries and NICs should cooperate closely in both monitoring health conditions and in fighting the pandemic through effective and efficient measures.
- Some of the NICs are active in well-established regional economic integration groups – in Asia it is ASEAN, in Latin America Mercosur, and in Africa the ECOWAS. Such existing regional economic cooperation networks could also be useful networks in terms of effectively fighting the pandemic. Strong regional integration links make increased cooperation in health systems and epidemic policy a natural international policy perspective.
- The EU, with its strong trade links to all three aforementioned regional integration clubs, could launch a broader inter-regional cooperation initiative in pandemic policy cooperation which could influence the G20 policy agenda. In a period of little evident US international leadership, inter-regional networking could become a more important pillar of new joint leadership, but institution building in the respective integration clubs should be sufficiently strong – for example, an effective supranational institution in is missing in both ASEAN and ECOWAS. Transaction costs for inter-regional cooperation could be expected to be rather modest if institutional setups are rather similar as is the case if one compares Mercosur and the EU27. There is, of course, the caveat that nationalist-populist policy approaches, such as that in Brazil under President Jair Bolsonaro, can be a formidable obstacle for such enhanced inter-regional cooperation.

In the end, the UN – along with the WHO – will have to play a strong role in getting the pandemic under full control worldwide. The UN could also have a special challenge in helping to avoid uncontrolled new international migration waves in the Corona Recession which could lead to a new broad spreading of the coronavirus.

Some of the key empirical findings about OECD countries are also valid for the NICs and the whole sample of countries, respectively. There are also differences across the two country groups. The overall picture is that demographic, medical, economic and environmental variables play a significant role. Moreover, the epidemic herd immunity strategy cannot be recommended as the COVID-19 fatality ratio is raised by such a policy approach (only in the case that no vaccination would be developed within 2020 could that strategy make sense). To the extent that countries’ governments do not want to implement a herd immunity strategy – allowing a controlled infection process and hoping that recovered infected patients become a critical barrier for spreading the virus – one would expect governments to implement anti-epidemic measures such as quarantine, social distancing and possibly lockdowns/shutdowns as an epidemic strategy while promoting R&D on new vaccines. A large number of clinical tests for new vaccines is expected to bring new insights and possibly a vaccination in 2021 for broader strata of global society. In any case it remains crucial to understand the drivers of infections and COVID-19 fatality and empirical research should help to shed light on the relevant dynamics.

The medium term overlap of medical and economic problems could be different in OECD and NICS, respectively. As regards OECD countries, both the output decline in the first quarter of 2020 and the size of the negative output forecast of the IMF in the June 2020 outlook (IMF 2020b) was larger than for NICs which roughly are composed of medium income and low income countries as defined by the World Bank. Several OECD countries have witnessed an appreciation of the currency in the first quarter of 2020 as high capital inflows from NICs were recorded, although the capital flow reversals were lower than in the Transatlantic Banking Crisis 2008/09: While NICs faced a strong currency depreciation after the US banking crisis in autumn 2008, the foreign exchange markets have reacted more modestly to the Corona shock in the first half year of 2020 (Esteves and Sussman 2020). This could mean that a rather limited economic shock in the South plus the rather rapid stabilization of China in the first half of 2020 could help the OECD countries to get their economies restarted in 2021.

An important conclusion from the findings presented herein is that a strategy of achieving herd immunity early on is doubtful as it raises the number of infections as well as the fatality ratio in a significant way; in a broader perspective this approach is less convincing the faster a vaccination against the coronavirus becomes available. While it is true that selective policy interventions – summarized in the mean policy stringency variable – is not significant in the regressions presented, it seems too early to discard the usefulness of such policy interventions which include social distancing and quarantine measures. There is likely an indirect effect in the form of a reduced number of cases of infection and this aspect, as well as questions of regional variations, could only be analyzed in further research. As regards the environmental air quality variable, one should emphasize two points here: (i) This variable should be carefully considered in order to anticipate particular regional/national epidemic hotspots in a future second infection wave. (ii) An emphasis on sustainability policies which bring down particulate matter intensities should be understood to be also part of strategic health care policy.

It is interesting to recall the British Government’s information on PM2.5, namely as noted by the British Department for Environment, Food & Rural Affairs on its website (HM Government 2020): “*Inhalation of particulate pollution can have adverse health impacts, and there is understood to be no safe threshold below which no adverse effects would be anticipated…The biggest impact of particulate air pollution on public health is understood to be from long-term exposure to PM2.5, which increases the age-specific mortality risk…*”. The government source continues to describe sources of PM2.5, in particular car traffic and industrial pollution, as well as heating processes; certain precursor gases are also relevant for the creation of PM2.5. In the future, assuming that our regression findings can be extended in a robust way for more UN countries – or a larger number of regions of the world economy - one would have to add the role of PM2.5 to an analysis of coronavirus pandemic fatality rates. One may expect that a switch from fossil fuels to renewable energy and climate change policy will considerably reduce PM2.5 air quality problems. According to the analysis presented herein, climate change policy would also reduce current and future fatality rates from COVID-19 and similar epidemics/pandemics so that there is an additional argument for promoting renewable energy and certain environmental innovations. The finding that obesity is a variable which is significantly raising case fatalities suggests that countries and regions, respectively, which have a relatively high indicator should prepare well for a second wave; and overlaps of regions showing high PM2.5 and high obesity indicators would suggest an “orange warning status”. The red warning status would be for those regions/countries where there is an overlap of high PM2.5, high obesity figures and a high share of elderly people in the overall population.

Given the nature of a pandemic and the potential cross-border diffusion of epidemics, respectively, it is clear that every national policy response and health system reform in OECD countries – as well as in other countries (assuming similar findings as in OECD countries) – has elements of a multi-country/global international public good. The economic logic thus suggests that countries should join forces in part of epidemic prevention health care expenditures. Particular attention should be paid to sharing the costs of anti-epidemic pharmaceutical and medical R&D. The OECD countries should come up with a new approach and a special funding agency here where the OECD’s outreach program – e.g. including non-member countries such as India and China – could be a starting point to also include some other countries in a strategic multilateral approach.

One may emphasize that the rather homogenous country group of OECD countries should find it easier to create an international health policy cooperation club with joint funding for international public goods than the economically much more heterogenous G20 group. To the extent that one ultimately wants to realize a global public good at the UN level – including all countries of the world – a lead initiative of the OECD could still be useful in order to generate sufficient momentum to achieve the provision of a global public good in a rather fast two-stage approach. A direct UN approach might also have some advantages, but there is a risk that heterogenous interests and the high number of countries involved would in the end mean a delayed provision of the global public good compared to the two-stage approach - or a three-stage approach: OECD-G20-UN (Welfens 2020b).

The fatality-increasing role of obesity points to a broad global need in the field of development policy not simply to push for an economic catching up of the global South which often goes along with a spreading of certain Western nutrition styles. Anti-obesity goals and an explicit emphasis on more sports activities for all generations as well enhanced company-based health and fitness programs should become a general element of catching-up policies. In the OECD countries themselves, policy initiatives for reducing obesity problems should follow a similar logic of better nutrition – such as encouraging the consumption of vegetables and fresh fruits as well as an emphasis, and more information, on low fat and low sugar products – and more sports. Institutionalized programs in schools, universities, the public administration and firms could be useful here, plus digital networking, which helps spreading relevant information and activities. The WHO has intensified its anti-obesity programs since 2018, but OECD countries have not been very active to include the relevant initiatives in its working programs: there is room for stronger WHO-OECD cooperation in this field and many OECD member countries, given high levels of obesity, have reason to become more active here.

Finally, the ageing of Western societies and of the population in Japan is a major long-term challenge for future epidemics. Beyond population policy and immigration incentives, little can be done in most OECD countries to slow the ageing process. However, there is an important policy implication with respect to membership contributions in certain international organizations. Given the international differential in terms of the ageing of populations of OECD countries (or UN member countries), one may argue that countries with a rather high ratio of the population aged 65 and over should contribute over-proportionately to the provision of international public goods in the field of prevention against and fighting of epidemics. So far in international organizations, the share of the elderly population plays no role in terms of the funding formula; the WHO could be the first organization where this aspect, emphasized in the research presented here, should have appropriate consequences. In a similar logic, one could argue that countries/regions with high PM2.5 indicators should also face higher contribution rates. The incentives from such modified contribution rates could clearly encourage welfare-enhancing political reforms and thus contribution formulas to international organizations could have a positive impact of global welfare in the long run. A broader analysis of UN countries is, however, required in a next empirical research step.

At the bottom line, it is clear that more research is needed, but the empirical findings presented could indeed be a useful starting point in the international economic and environmental coronavirus research. The broader research challenges in many ways will also require enhanced interdisciplinary research which would, of course, include the medical sciences on many topics. Both internationally comparative research, regional analysis, as well as spatial regression analysis for cities could be crucial – see, for example, for New York (Chen et al. 2020); among the findings for New York, using spatial regression analysis, one may note that many contact-intensification points, including grocery shop density, green space density and median distance travelled plus, paradoxically, POIs of medicine density turned out to have a positive significant impact on infections. In a more international view, intensive contacts through travelling – possibly related to trade, foreign investment or tourism – could be critical epidemic diffusion points which could indicate that the shadow price of economic globalization might be higher than traditionally considered. In a nutshell, the urban centers of globalization around the world could pay a higher price in a COVID-19 environment than less densely populated cities, regions and countries.

Here, and in the internationally comparative environmental quality dimensions, much future coronavirus research could be expected. As regards conclusions for policymakers, the suggested implications of our regression findings for dealing with a potential second wave of infections are already highly sensitive to being picked up quickly in the public debate worldwide.

The table below shows the number of lives which could have been saved had the spread of COVID-19 infections been delayed by 7 days and 14 days, respectively: Looking at the case of a 14-day delay, 37 countries would have saved more than 1,000 lives where the worst performers in absolute terms, namely India, the US and Indonesia, stand for approximately 60,000, 14,000 and 12,000 deaths which could have been avoided, respectively; if one considers the case of a 7-day delay, there are still 25 countries which each could have saved a minimum of 1,000 lives each. For the three worst countries, the sum of lives saved would have been 42,814 (7-day delay) and 85,629 (14-day delay), for the 20 worst countries – coming both from OECD countries and NICs/LDCs – the sum would have been 83,460 (7-day delay) and 166,920 (14-day delay). The grand total for 104 countries would have been 108,185 (7-day delay) and 216,369 (14-day delay), respectively. The 7-day delay figure is a hypothetical implicit finding based on the present study and in most industrialized countries the number of lives saved from postponing the first infections by one week indeed makes sense (e.g., 5% in the US; 5% in France, 4% in Italy and Spain, 9% in Canada – based on total COVID-19 fatalities recorded as of July 20, 2020). In countries with very low number of fatalities officially recorded – often obviously standing for underreporting in many developing countries – or in economies where the peak of the infection is still several months away, the implicit figure for lives saved as a result of a 7-day delay could exceed the actual number of fatalities by the reference date of July 20. However, the approach itself remains a useful analytical exercise and by the end of 2020 more countries with meaningful (post-peak) results should be also be recorded in the so-called South of the world economy. Lower fatality rates typically mean less problems for the relevant health care system and certainly also welfare gains; in terms of the economic effects, a lower fatality figure could not only mean higher effective employment figures in the medium term and long run but also less of a negative psychological “scarring effect” in the population which undermines consumption demand. Countries with a relatively early and focused epidemic policy have thus recorded considerable gains by saving lives. Countries with greatly reduced fatality figures (in the case of delaying infections) from the same region would have benefitted jointly from a lower reduction of trade, foreign investment and output if one follows the logic of the gravity equation for trade and foreign direct investment.

## Appendix

**Table 9:** Correlation Matrix

Variables	(1)	(2)	(3)	(4)	(5)	(6)	(7)	(8)	(9)	(10)	(11)
(1) Deaths per million	1.00										
(2) Cases per million	0.48*	1.00									
(3) Average growth rate of deaths	0.27*	0.11	1.00								
(4) Average growth rate of cases	0.32*	0.43*	0.51*	1.00							
(5) PM2.5 in largest FUA	−0.03	−0.02	−0.06	0.14	1.00						
(6) PM2.5 air pollution, mean annual exposure	−0.25*	0.25*	0.06	0.22*	0.95*	1.00					
(7) PM2.5 in most populous city available	−0.27*	0.09	0.04	0.14	0.88*	0.77*	1.00				
(8) Percent over 65, largest FUA	−0.22	−0.34	−0.16	−0.15	−0.03	−0.13	−0.08	1.00			
(9) Median age	0.30*	0.07	−0.03	−0.11	−0.02	−0.41*	−0.43*	0.89*	1.00		
(10) Percent overweight	0.39*	0.35*	0.08	0.28*	−0.24	−0.30*	−0.39*	−0.17	0.57*	1.00	
(11) Percent smokers	0.10	−0.10	0.09	−0.09	0.36*	−0.18	−0.08	0.32	0.53*	0.30*	1.00
(12) Diabetes prevalence	−0.15	0.21*	−0.22*	0.00	0.23	0.27*	0.36*	−0.19	0.04	0.23*	−0.07
(13) Death rate from CVD	−0.37*	−0.25*	0.03	−0.07	0.14	0.24*	0.33*	0.17	−0.27*	−0.18*	0.20*
(14) Hospital beds/thousand	0.01	−0.13	0.02	−0.13	0.31	−0.30*	−0.22	0.38*	0.64*	0.21*	0.49*
(15) GHS index	0.31*	0.08	0.41*	0.14	−0.38*	−0.33*	−0.38*	−0.18	0.66*	0.38*	0.26*
(16) Days from Jan 1 until first case	−0.25*	−0.09	0.00	0.04	0.18	−0.01	0.13	−0.06	−0.44*	−0.07	−0.05
(17) Herd immunity policy	0.36*	0.11	0.23*	0.09	−0.20	−0.14	−0.16	−0.12	0.13	0.12	−0.04
(18) Days until travel restrictions, from 10 cases	0.31*	0.12	−0.02	0.03	−0.16	−0.21*	−0.26*	−0.27	0.38*	0.39*	0.07
(19) Mean policy stringency	−0.01	0.16	0.12	0.15	0.37*	0.29*	0.46*	−0.17	−0.18*	−0.01	−0.11
(20) GDP per capita	0.31*	0.55*	−0.08	0.12	−0.51*	−0.11	−0.31*	−0.17	0.56*	0.46*	0.05
(21) Population density	−0.03	0.16	−0.14	−0.12	0.32	0.05	0.05	0.12	0.18*	−0.14	−0.07
Variables	(12)	(13)	(14)	(15)	(16)	(17)	(18)	(19)	(20)	(21)	
(12) Diabetes prevalence	1.00										
(13) Death rate from CVD	0.03	1.00									
(14) Hospital beds/thousand	−0.20*	0.09	1.00								
(15) GHS index	−0.14	−0.38*	0.28*	1.00							
(16) Days from Jan 1 until first case	0.02	0.26*	−0.19*	−0.61*	1.00						
(17) Herd immunity policy	−0.11	−0.15	−0.06	0.33*	−0.14	1.00					
(18) Days until travel restrictions, from 10 cases	−0.02	−0.24*	0.12	0.36*	−0.30*	0.27*	1.00				
(19) Mean policy stringency	0.17	0.20*	−0.17	−0.12	0.03	−0.09	−0.24*	1.00			
(20) GDP per capita	0.20*	−0.44*	0.21*	0.46*	−0.35*	0.11	0.33*	−0.14	1.00		
(21) Population density	0.13	−0.16	−0.03	0.06	−0.14	−0.02	−0.02	0.07	0.32*	1.00	

*shows significance at the .05 level

**Table 10:** Sample Selection

Num	NAMES_STD	ISO3	in_32	in_37	in_75	in_77	in_98	in_104	in_106	in_108
1	Albania	ALB	No	No	Yes	Yes	Yes	Yes	Yes	Yes
2	United Arab Emirates	ARE	No	No	No	Yes	Yes	Yes	Yes	Yes
3	Argentina	ARG	No	No	No	Yes	Yes	Yes	Yes	Yes
4	Australia	AUS	Yes	Yes	Yes	Yes	Yes	Yes	Yes	Yes
5	Austria	AUT	Yes	Yes	Yes	Yes	Yes	Yes	Yes	Yes
6	Azerbaijan	AZE	No	No	No	Yes	Yes	Yes	Yes	Yes
7	Belgium	BEL	Yes	Yes	Yes	Yes	Yes	Yes	Yes	Yes
8	Benin	BEN	No	No	No	No	Yes	Yes	Yes	Yes
9	Bangladesh	BGD	No	No	Yes	No	Yes	Yes	Yes	Yes
10	Bulgaria	BGR	No	No	Yes	Yes	Yes	Yes	Yes	Yes
11	Bahrain	BHR	No	No	Yes	Yes	Yes	Yes	Yes	Yes
12	Bosnia and Herzegovina	BIH	No	No	Yes	Yes	Yes	Yes	Yes	Yes
13	Brazil	BRA	No	No	Yes	Yes	Yes	Yes	Yes	Yes
14	Barbados	BRB	No	No	No	Yes	Yes	Yes	Yes	Yes
15	Brunei Darussalam	BRN	No	No	No	Yes	Yes	Yes	Yes	Yes
16	Botswana	BWA	No	No	No	Yes	Yes	Yes	Yes	Yes
17	Canada	CAN	Yes	Yes	Yes	Yes	Yes	Yes	Yes	Yes
18	Switzerland	CHE	Yes	Yes	Yes	Yes	Yes	Yes	Yes	Yes
19	Chile	CHL	Yes	Yes	Yes	Yes	Yes	Yes	Yes	Yes
20	China	CHN	No	No	No	No	No	No	No	Yes
21	Colombia	COL	No	Yes	Yes	Yes	Yes	Yes	Yes	Yes
22	Cabo Verde	CPV	No	No	No	No	Yes	Yes	Yes	Yes
23	Costa Rica	CRI	No	No	Yes	Yes	Yes	Yes	Yes	Yes
24	Cuba	CUB	No	No	Yes	Yes	Yes	Yes	No	Yes
25	Cyprus	CYP	No	No	Yes	Yes	Yes	Yes	Yes	Yes
26	Czech Republic	CZE	Yes	Yes	Yes	Yes	Yes	Yes	Yes	Yes
27	Germany	DEU	Yes	Yes	Yes	Yes	Yes	Yes	Yes	Yes
28	Djibouti	DJI	No	No	No	No	Yes	Yes	Yes	Yes
29	Denmark	DNK	Yes	Yes	Yes	Yes	Yes	Yes	Yes	Yes
30	Dominican Republic	DOM	No	No	No	Yes	Yes	Yes	Yes	Yes
31	Algeria	DZA	No	No	No	No	Yes	Yes	Yes	Yes
32	Ecuador	ECU	No	No	Yes	Yes	Yes	Yes	Yes	Yes
33	Egypt, Arab Rep.	EGY	No	No	No	No	Yes	Yes	Yes	Yes
34	Spain	ESP	Yes	Yes	Yes	Yes	Yes	Yes	Yes	Yes
35	Estonia	EST	Yes	Yes	Yes	Yes	Yes	Yes	Yes	Yes
36	Finland	FIN	Yes	Yes	Yes	Yes	Yes	Yes	Yes	Yes
37	Fiji	FJI	No	No	Yes	Yes	No	Yes	Yes	Yes
38	France	FRA	Yes	Yes	Yes	Yes	Yes	Yes	Yes	Yes
39	United Kingdom	GBR	Yes	Yes	Yes	Yes	Yes	Yes	Yes	Yes
40	Georgia	GEO	No	No	Yes	Yes	Yes	Yes	Yes	Yes
41	Ghana	GHA	No	No	Yes	No	Yes	Yes	Yes	Yes
42	Greece	GRC	Yes	Yes	Yes	Yes	Yes	Yes	Yes	Yes
43	Croatia	HRV	No	No	Yes	Yes	Yes	Yes	Yes	Yes
44	Hungary	HUN	Yes	Yes	Yes	Yes	Yes	Yes	Yes	Yes
45	Indonesia	IDN	No	No	Yes	Yes	Yes	Yes	Yes	Yes
46	India	IND	No	No	Yes	No	Yes	Yes	Yes	Yes
47	Ireland	IRL	Yes	Yes	Yes	Yes	Yes	Yes	Yes	Yes
48	Iran, Islamic Rep.	IRN	No	No	Yes	Yes	Yes	Yes	Yes	Yes
49	Iceland	ISL	No	Yes	Yes	Yes	Yes	Yes	Yes	Yes
50	Israel	ISR	No	Yes	Yes	Yes	Yes	Yes	Yes	Yes
51	Italy	ITA	Yes	Yes	Yes	Yes	Yes	Yes	Yes	Yes
52	Jamaica	JAM	No	No	No	Yes	Yes	Yes	Yes	Yes
53	Japan	JPN	Yes	Yes	Yes	Yes	Yes	Yes	Yes	Yes
54	Kazakhstan	KAZ	No	No	No	Yes	Yes	Yes	Yes	Yes
55	Kenya	KEN	No	No	Yes	No	Yes	Yes	Yes	Yes
56	Kyrgyz Republic	KGZ	No	No	No	No	Yes	Yes	Yes	Yes
57	Cambodia	KHM	No	No	No	No	No	Yes	Yes	Yes
58	Korea, Rep.	KOR	Yes	Yes	Yes	Yes	Yes	Yes	Yes	Yes
59	Kuwait	KWT	No	No	Yes	Yes	Yes	Yes	Yes	Yes
60	Lao PDR	LAO	No	No	No	No	No	Yes	Yes	Yes
61	Sri Lanka	LKA	No	No	No	No	Yes	Yes	Yes	Yes
62	Lesotho	LSO	No	No	No	No	No	No	Yes	Yes
63	Lithuania	LTU	Yes	Yes	Yes	Yes	Yes	Yes	Yes	Yes
64	Luxembourg	LUX	Yes	Yes	Yes	Yes	Yes	Yes	Yes	Yes
65	Latvia	LVA	Yes	Yes	Yes	Yes	Yes	Yes	Yes	Yes
66	Morocco	MAR	No	No	Yes	No	Yes	Yes	Yes	Yes
67	Moldova	MDA	No	No	No	No	Yes	Yes	Yes	Yes
68	Mexico	MEX	Yes	Yes	Yes	Yes	Yes	Yes	Yes	Yes
69	Mongolia	MNG	No	No	Yes	No	No	Yes	Yes	Yes
70	Mauritius	MUS	No	No	No	Yes	Yes	Yes	Yes	Yes
71	Malaysia	MYS	No	No	No	Yes	Yes	Yes	Yes	Yes
72	Namibia	NAM	No	No	No	No	No	No	Yes	Yes
73	Netherlands	NLD	Yes	Yes	Yes	Yes	Yes	Yes	Yes	Yes
74	Norway	NOR	Yes	Yes	Yes	Yes	Yes	Yes	Yes	Yes
75	Nepal	NPL	No	No	Yes	No	Yes	Yes	Yes	Yes
76	New Zealand	NZL	No	Yes	Yes	Yes	Yes	Yes	Yes	Yes
77	Oman	OMN	No	No	No	Yes	Yes	Yes	Yes	Yes
78	Pakistan	PAK	No	No	Yes	No	Yes	Yes	Yes	Yes
79	Panama	PAN	No	No	Yes	Yes	Yes	Yes	Yes	Yes
80	Philippines	PHL	No	No	Yes	No	Yes	Yes	Yes	Yes
81	Poland	POL	Yes	Yes	Yes	Yes	Yes	Yes	Yes	Yes
82	Portugal	PRT	Yes	Yes	Yes	Yes	Yes	Yes	Yes	Yes
83	Paraguay	PRY	No	No	Yes	Yes	Yes	Yes	Yes	Yes
84	Qatar	QAT	No	No	No	Yes	Yes	Yes	Yes	Yes
85	Romania	ROU	No	No	Yes	Yes	Yes	Yes	Yes	Yes
86	Russian Federation	RUS	No	No	Yes	Yes	Yes	Yes	Yes	Yes
87	Saudi Arabia	SAU	No	No	Yes	Yes	Yes	Yes	Yes	Yes
88	Senegal	SEN	No	No	No	No	No	No	Yes	Yes
89	Singapore	SGP	No	No	Yes	Yes	Yes	Yes	Yes	Yes
90	El Salvador	SLV	No	No	Yes	No	Yes	Yes	Yes	Yes
91	Serbia	SRB	No	No	Yes	Yes	Yes	Yes	Yes	Yes
92	Suriname	SUR	No	No	No	Yes	Yes	Yes	Yes	Yes
93	Slovak Republic	SVK	Yes	Yes	Yes	Yes	Yes	Yes	Yes	Yes
94	Slovenia	SVN	Yes	Yes	Yes	Yes	Yes	Yes	Yes	Yes
95	Sweden	SWE	Yes	Yes	Yes	Yes	Yes	Yes	Yes	Yes
96	Eswatini	SWZ	No	No	No	No	Yes	Yes	Yes	Yes
97	Seychelles	SYC	No	No	No	Yes	No	Yes	Yes	Yes
98	Thailand	THA	No	No	Yes	Yes	Yes	Yes	Yes	Yes
99	Tunisia	TUN	No	No	No	No	Yes	Yes	Yes	Yes
100	Turkey	TUR	No	Yes	Yes	Yes	Yes	Yes	Yes	Yes
101	Tanzania	TZA	No	No	Yes	No	Yes	Yes	Yes	Yes
102	Ukraine	UKR	No	No	Yes	No	Yes	Yes	Yes	Yes
103	Uruguay	URY	No	No	Yes	Yes	Yes	Yes	Yes	Yes
104	United States	USA	Yes	Yes	Yes	Yes	Yes	Yes	Yes	Yes
105	Uzbekistan	UZB	No	No	No	No	Yes	Yes	Yes	Yes
106	Vietnam	VNM	No	No	Yes	No	No	Yes	Yes	Yes
107	South Africa	ZAF	No	No	Yes	Yes	Yes	Yes	Yes	Yes
108	Zambia	ZMB	No	No	No	No	Yes	Yes	Yes	Yes

**Table 11:** Robustness check, Full sample, COVID-19 Deaths

Variables	(1)	(2)	(3)	(4)	(5)
	Deaths per million	Deaths per million	Deaths per million	Deaths per million	Avg. growth rate, deaths
	full sample	full sample	full sample	U. middle & High income	full sample
PM2.5 air pollution, mean annual exposure	−0.817	−0.951		−1.824	0.022*
	(0.865)	(0.780)		(1.759)	(0.013)
PM2.5 in most populous city available			0.586		
			(0.878)		
Cases per million	0.010**	0.008**	0.014***	0.009**	
	(0.005)	(0.004)	(0.004)	(0.004)	
Median age	−2.497	−6.288**	−8.914**	−6.320*	−0.163***
	(2.713)	(2.801)	(3.972)	(3.409)	(0.059)
Percent overweight	1.148	1.905	4.509*	3.266	0.011
	(1.111)	(1.415)	(2.403)	(2.312)	(0.029)
Percent smokers	0.041	1.608	1.265	1.757	0.053
	(1.806)	(1.695)	(2.220)	(2.532)	(0.035)
Death rate from CVD	−0.373***	−0.211*	−0.152	−0.393**	−0.00004
	(0.132)	(0.124)	(0.162)	(0.178)	(0.002)
Diabetes prevalence	−5.964	−2.508	−7.708	1.480	−0.056
	(3.722)	(3.472)	(7.549)	(4.226)	(0.055)
GHS index		0.345	0.933	−0.774	0.084***
		(1.569)	(2.479)	(2.442)	(0.027)
Herd immunity policy		237.469***	186.228***	244.461***	1.672*
		(54.260)	(58.452)	(57.459)	(0.957)
Days until travel restrictions, from 10 cases		0.505	0.530		
		(0.372)	(0.520)		
Days from Jan 1 until first case		−2.911**	−4.486**	−4.797**	−0.036**
		(1.357)	(2.035)	(2.014)	(0.017)
Constant	191.999*	304.602*	303.668	365.840	5.040*
	(100.302)	(159.503)	(226.017)	(255.648)	(2.788)
Observations	108	106	76	78	100
R-squared	0.397	0.572	0.624	0.583	0.413
Region dummies	YES	YES	YES	YES	YES

Robust standard errors in parentheses

*** p < 0.01, ** p < 0.05, * p < 0.1

**Table 12:** Robustness check, Full sample, COVID-19 Cases

Variables	(1)	(2)	(3)	(4)	(5)
	Cases per million	Cases per million	Cases per million	Cases per million	Avg. growth rate, cases
	full sample	full sample	full sample	U. middle & High income	full sample
PM2.5 air pollution, mean annual exposure	82.237*	82.532*		153.784*	0.041**
	(45.554)	(47.970)		(85.986)	(0.018)
PM2.5 in most populous city available			42.716**		
			(16.972)		
Median age	−135.163**	−125.666*	−93.550	−152.123	−0.118*
	(63.048)	(74.144)	(80.861)	(95.558)	(0.065)
Percent overweight	−15.582	−17.510	37.518	35.932	0.021
	(31.844)	(38.779)	(26.623)	(45.396)	(0.030)
Percent smokers	53.914	60.324	158.671	96.815	−0.008
	(73.994)	(79.847)	(96.707)	(106.404)	(0.031)
Death rate from CVD	0.538	−0.498	−2.682	−0.715	−0.001
	(4.698)	(4.849)	(4.974)	(6.496)	(0.003)
Diabetes prevalence	10.242	−10.516	323.789*	−91.113	−0.004
	(107.408)	(104.436)	(161.754)	(142.934)	(0.065)
GHS index	24.107	4.411	−45.980	−1.777	0.055*
	(45.340)	(46.341)	(51.891)	(55.485)	(0.029)
GDP per capita	0.158***	0.158***	0.120***	0.144***	0.00002
	(0.050)	(0.057)	(0.030)	(0.050)	(0.000)
Population density		0.155	0.290	0.256	0.00004
		(0.611)	(0.424)	(0.468)	(0.000)
Herd immunity policy		3397.35*	3851.24**	3676.91*	−0.08
		(1930.06)	(1525.08)	(1852.67)	(1.04)
Days until travel restrictions, from 10 cases		−23.760*	−10.142	−19.548	−0.004
		(12.685)	(11.233)	(12.224)	(0.007)
Days from Jan 1 until first case		−10.913	−68.604***	−35.085	0.007
		(27.827)	(22.915)	(37.411)	(0.020)
Constant	−2753.39	−1801.01	−2511.10	−2843.47	4.71
	(4369.65)	(4549.43)	(5746.17)	(7834.86)	(3.04)
Observations	106	105	75	77	105
R-squared	0.572	0.599	0.654	0.683	0.411
Region dummies	YES	YES	YES	NO	YES

Robust standard errors in parentheses

*** p < 0.01, ** p < 0.05, * p < 0.1

**Table 13:** Lives Saved by Postponing COVID-19 Infections by 7 Days/14 Days (based on Tab. 7). The table below shows the number of lives which could have been saved had the spread of COVID-19 infections been delayed by 7 days and 14 days, respectively: Looking at the case of a 14-day delay, 37 countries would have saved more than 1000 lives where the worst performers in absolute terms, namely India, the US and Indonesia, stand for approximately 60,000, 14,000 and 12,000 deaths which could have been avoided, respectively; if one considers the case of a 7-day delay, there are still 25 countries which each could have saved a minimum of 1000 lives each. For the three worst countries, the sum of lives saved would have been 42,814 (7-day delay) and 85,629 (14-day delay), for the 20 worst countries – coming both from OECD countries and NICs/LDCs – the sum would have been 83,460 (7-day delay) and 166,920 (14-day delay). The grand total for 104 countries would have been 108,185 (7-day delay) and 216,369 (14-day delay), respectively. The 7-day delay figure is a hypothetical implicit finding based on the present study and in most industrialized countries the number of lives saved from postponing the first infections by one week indeed makes sense (e.g., 5% in the US; 5% in France, 4% in Italy and Spain, 9% in Canada – based on total COVID-19 fatalities recorded as of July 20, 2020). In countries with very low number of fatalities officially recorded – often obviously standing for underreporting in many developing countries – or in economies where the peak of the infection is still several months away, the implicit figure for lives saved as a result of a 7-day delay could exceed the actual number of fatalities by the reference date of July 20. However, the approach itself remains a useful analytical exercise and by the end of 2020 more countries with meaningful (post-peak) results should be also be recorded in the so-called South of the world economy. Lower fatality rates typically mean less problems for the relevant health care system and certainly also welfare gains; in terms of the economic effects, a lower fatality figure could not only mean higher effective employment figures in the medium term and long run but also less of a negative psychological “scarring effect” in the population which undermines consumption demand. Countries with a relatively early and focused epidemic policy have thus recorded considerable gains by saving lives. Countries with greatly reduced fatality figures (in the case of delaying infections) from the same region would have benefitted jointly from a lower reduction of trade, foreign investment and output if one follows the logic of the gravity equation for trade and foreign direct investment.

No.	ISO3	Economies	a) Population	b) 7 days delay figure	c) 14 days delay figure	d) COVID-19 Total deaths until 20th July 2020
1	IND	India	1,380,004,385	29,772.21	59,544.43	27,497
2	USA	United States	331,002,647	7141.05	14,282.10	140,534
3	IDN	Indonesia	273,523,621	5901.00	11,802.00	4143
4	PAK	Pakistan	220,892,331	4765.53	9531.06	5599
5	BRA	Brazil	212,559,409	4585.76	9171.51	79,488
6	BGD	Bangladesh	164,689,383	3553.01	7106.02	2618
7	RUS	Russian Federation	145,934,460	3148.39	6296.78	12,342
8	MEX	Mexico	128,932,753	2781.60	5563.19	39,184
9	JPN	Japan	126,476,458	2728.60	5457.21	985
10	PHL	Philippines	109,581,085	2364.10	4728.20	1831
11	EGY	Egypt, Arab Rep.	102,334,403	2207.76	4415.52	4302
12	VNM	Vietnam	97,338,583	2099.98	4199.97	0
13	TUR	Turkey	84,339,067	1819.53	3639.06	5491
14	IRN	Iran, Islamic Rep.	83,992,953	1812.06	3624.13	14,188
15	DEU	Germany	83,783,945	1807.55	3615.11	9086
16	THA	Thailand	69,799,978	1505.86	3011.73	58
17	GBR	United Kingdom	67,886,004	1464.57	2929.15	45,300
18	FRA	France	65,273,512	1408.21	2816.42	30,152
19	ITA	Italy	60,461,828	1304.40	2608.81	35,045
20	TZA	Tanzania	59,734,213	1288.71	2577.41	21
21	ZAF	South Africa	59,308,690	1279.53	2559.05	5033
22	KEN	Kenya	53,771,300	1160.06	2320.12	234
23	KOR	Korea, Rep.	51,269,183	1106.08	2212.16	296
24	COL	Colombia	50,882,884	1097.75	2195.49	6736
25	ESP	Spain	46,754,783	1008.69	2017.38	28,422
26	ARG	Argentina	45,195,777	975.05	1950.11	2246
27	DZA	Algeria	43,851,043	946.04	1892.08	1078
28	UKR	Ukraine	43,733,759	943.51	1887.02	1485
29	POL	Poland	37,846,605	816.50	1633.01	1624
30	CAN	Canada	37,742,157	814.25	1628.50	8852
31	MAR	Morocco	36,910,558	796.31	1592.62	273
32	SAU	Saudi Arabia	34,813,867	751.07	1502.15	2486
33	UZB	Uzbekistan	33,469,199	722.06	1444.13	88
34	MYS	Malaysia	32,365,998	698.26	1396.53	123
35	GHA	Ghana	31,072,945	670.37	1340.74	148
36	NPL	Nepal	29,136,808	628.60	1257.19	40
37	AUS	Australia	25,499,881	550.13	1100.27	122
38	LKA	Sri Lanka	21,413,250	461.97	923.94	11
39	ROU	Romania	19,237,682	415.03	830.07	2026
40	CHL	Chile	19,116,209	412.41	824.83	8503
41	KAZ	Kazakhstan	18,776,707	405.09	810.18	375
42	ZMB	Zambia	18,383,956	396.62	793.23	109
43	ECU	Ecuador	17,643,060	380.63	761.26	5313
44	NLD	Netherlands	17,134,873	369.67	739.34	6136
45	KHM	Cambodia	16,718,971	360.70	721.39	0
46	BEN	Benin	12,123,198	261.55	523.09	31
47	TUN	Tunisia	11,818,618	254.97	509.95	50
48	BEL	Belgium	11,589,616	250.03	500.07	9800
49	CUB	Cuba	11,326,616	244.36	488.72	87
50	DOM	Dominican Republic	10,847,904	234.03	468.07	981
51	CZE	Czech Republic	10,708,982	231.04	462.07	359
52	GRC	Greece	10,423,056	224.87	449.73	194
53	PRT	Portugal	10,196,707	219.98	439.97	1689
54	AZE	Azerbaijan	10,139,175	218.74	437.49	354
55	SWE	Sweden	10,099,270	217.88	435.76	5619
56	ARE	United Arab Emirates	9,890,400	213.38	426.75	339
57	HUN	Hungary	9,660,350	208.41	416.82	596
58	AUT	Austria	9,006,400	194.30	388.61	711
59	ISR	Israel	8,655,541	186.73	373.47	409
60	CHE	Switzerland	8,654,618	186.71	373.43	1687
61	LAO	Lao PDR	7,275,556	156.96	313.93	0
62	PRY	Paraguay	7,132,530	153.88	307.75	31
63	BGR	Bulgaria	6,948,445	149.91	299.81	300
64	SRB	Serbia	6,804,596	146.80	293.60	472
65	KGZ	Kyrgyz Republic	6,524,191	140.75	281.51	1037
66	SLV	El Salvador	6,486,201	139.93	279.87	344
67	SGP	Singapore	5,850,343	126.22	252.43	27
68	DNK	Denmark	5,792,203	124.96	249.92	611
69	FIN	Finland	5,540,718	119.54	239.07	328
70	SVK	Slovak Republic	5,459,643	117.79	235.57	28
71	NOR	Norway	5,421,242	116.96	233.92	255
72	OMN	Oman	5,106,622	110.17	220.34	318
73	CRI	Costa Rica	5,094,114	109.90	219.80	62
74	IRL	Ireland	4,937,796	106.53	213.06	1753
75	NZL	New Zealand	4,822,233	104.03	208.07	22
76	PAN	Panama	4,314,768	93.09	186.17	1096
77	KWT	Kuwait	4,270,563	92.13	184.27	408
78	HRV	Croatia	4,105,268	88.57	177.13	120
79	MDA	Moldova	4,033,963	87.03	174.06	684
80	GEO	Georgia	3,989,175	86.06	172.12	15
81	URY	Uruguay	3,473,727	74.94	149.88	33
82	BIH	Bosnia and Herzegovina	3,280,815	70.78	141.56	245
83	MNG	Mongolia	3,278,292	70.73	141.45	0
84	JAM	Jamaica	2,961,161	63.88	127.77	10
85	QAT	Qatar	2,881,060	62.16	124.31	157
86	ALB	Albania	2,877,800	62.09	124.17	112
87	LTU	Lithuania	2,722,291	58.73	117.46	80
88	BWA	Botswana	2,351,625	50.73	101.47	1
89	SVN	Slovenia	2,078,932	44.85	89.70	111
90	LVA	Latvia	1,886,202	40.69	81.39	31
91	BHR	Bahrain	1,701,583	36.71	73.42	126
92	EST	Estonia	1,326,539	28.62	57.24	69
93	MUS	Mauritius	1,271,767	27.44	54.87	10
94	SWZ	Eswatini	1160,164	25.03	50.06	21
95	DJI	Djibouti	988,002	21.32	42.63	56
96	FJI	Fiji	896,444	19.34	38.68	0
97	CYP	Cyprus	875,899	18.90	37.79	19
98	LUX	Luxembourg	625,976	13.50	27.01	111
99	SUR	Suriname	586,634	12.66	25.31	21
100	CPV	Cabo Verde	555,988	11.99	23.99	21
101	BRN	Brunei Darussalam	437,483	9.44	18.88	3
102	ISL	Iceland	341,250	7.36	14.72	10
103	BRB	Barbados	287,371	6.20	12.40	7
104	SYC	Seychelles	98,340	2.12	4.24	0
Grand total			5,014,587,129	108,185	216,369	571,694

Note: One unit of days from Jan 1 until first case increase leads to 3.082 units of deaths per million increase based on the sample of 104 countries in Table 7, the calculation formula is: 3.082/million*days delay*population

## Footnotes

1 In January 2020, the World Health Organization (WHO) declared that the COVID-19 outbreak met the criteria to be classified as a “Public Health Emergency of International Concern” (WHO 2020b). On March 11, 2020, the WHO finally declared the international epidemic to be a pandemic, namely an epidemic which was now affecting countries in all regions of the world (WHO 2020c).

2 Some German hospitals could accommodate a relatively small number of COVID-19 patients from Italy and France in April 2020. While the anti-epidemic policy measures in OECD countries have helped to bring down infection rates and to flatten the infection and case fatality curves over time, the cumulated number of COVID-19 fatalities in some EU countries have been rather high – for example, in the UK, Italy and Spain - while Germany has recorded a rather low number of case fatalities.

3 A specific problem concerns how COVID-19 case fatalities are classified where death cases in care homes presents a particular issue – the relative number of case fatalities seems to be relatively large as the elderly have higher death rates than the younger generation; in particular, the identification of a case of COVID-19 in a care home for the elderly in Belgium has the consequence all further death cases in that care home in spring and early summer 2020 were automatically classified as COVID-19 cases without further testing. Different coverage of testing across countries – including post-mortem testing – thus lead to different numbers of case fatalities.

4 It cannot be ruled out that after an initial phase of flattening the infection and death curves, there could be a second wave of infection and, in the future, a third wave of infection - until either a vaccine is available or herd immunity is achieved.

5 The new pandemic is creating enormous challenges in OECD countries and the uncertainty in the early months of that pandemic makes determining adequate policy measures aimed at fighting the pandemic a difficult task: It seems that most OECD countries did not have adequate stocks of masks, disinfectants and medical personal protective equipment for the coronavirus pandemic, despite the fact that, e.g., all EU countries and Switzerland had an established official pandemic plan. Indeed, the pandemic quickly revealed weak points in many OECD countries.

6 The contribution discusses the tradable and the non-tradable sectors and considers the role of international tourism as well as some growth modelling insights (with effective labor supply in the production function negatively affected by the share of uninsured population/workers with a weaker health status). Moreover, with respect to a potential corona morbidity risk, the ratio of acute care beds relative to the population aged 65 and above is emphasized, as it seems that fatality rates are higher for the elderly.

7 The model assumes that labor input in the production function is negatively influenced by infections and COVID-19 death cases, respectively - so that welfare analysis can be applied within a hybrid economic-epidemic approach.

8 It is noteworthy that an online, regional COVID-19 Simulator tool was quickly developed by two research groups (Harvard Medical School researchers based at Massachusetts General Hospital and researchers from the Georgia Institute of Technology) which allows an understanding of the impact of alternative regional policy strategies in terms of soft measures versus stricter regional lockdowns.

9 In the EuroMOMO (2020a) Bulletin of week 18, 2020, key findings are summarized as follows: (i) “...overall excess mortality is driven by a very substantial excess mortality in some countries, while other countries have had no excess mortality. The mortality excess is primarily seen in the age group of > = 65 years, but also in the age group of 15–64 years” and (ii) the EuroMOMO (2020b) Bulletin of week 19 shows that England had the highest excess mortality in week 17, 2020, while Germany, for example – actually Berlin and Hesse as two possibly representative German states – showed no excess mortality in the whole first quarter of 2020. Germany officially had about 7000 Corona case fatalities by late April. This makes clear that replacing WHO data by excess mortality figures also can have its problems. The Italian statistical office (ISTAT 2020) has calculated regional excess case fatalities which, unsurprisingly, show considerable variation across regions.

10 The UK is an interesting case since the coverage in Scotland, for example, in March 2020 was broader than that in England and Wales.

11 Manski and Molinari (2020) highlight the absence of bounds on infection rates and explain the logical problem of bounding them; they find that the actual infection rates might be substantially higher than reported.

12 The definition of a COVID-19 fatality according to the WHO (2020d) is as follows: *“COVID-19 death is defined for surveillance purposes as a death resulting from a clinically compatible illness in a probable or confirmed COVID-19 case, unless there is a clear alternative cause of death that cannot be related to COVID disease (*e.g. *trauma). There should be no period of complete recovery between the illness and death.”*
